# Recent Progress in Biosensors for Depression Monitoring—Advancing Personalized Treatment

**DOI:** 10.3390/bios14090422

**Published:** 2024-08-30

**Authors:** Jiaju Yin, Xinyuan Jia, Haorong Li, Bingchen Zhao, Yi Yang, Tian-Ling Ren

**Affiliations:** 1 School of Integrated Circuits, Tsinghua University, Beijing 100084, China; yjj22@mails.tsinghua.edu.cn (J.Y.); zhaobc22@mails.tsinghua.edu.cn (B.Z.); 2 Beijing National Research Center for Information Science and Technology (BNRist), Tsinghua University, Beijing 100084, China; 3 Xingjian College, Tsinghua University, Beijing 100084, China; jia-xy21@mails.tsinghua.edu.cn; 4 Weiyang College, Tsinghua University, Beijing 100084, China; lihr22@mails.tsinghua.edu.cn; 5 Center for Flexible Electronics Technology, Tsinghua University, Beijing 100084, China

**Keywords:** biosensors, depression, personalized treatment

## Abstract

Depression is currently a major contributor to unnatural deaths and the healthcare burden globally, and a patient’s battle with depression is often a long one. Because the causes, symptoms, and effects of medications are complex and highly individualized, early identification and personalized treatment of depression are key to improving treatment outcomes. The development of wearable electronics, machine learning, and other technologies in recent years has provided more possibilities for the realization of this goal. Conducting regular monitoring through biosensing technology allows for a more comprehensive and objective analysis than previous self-evaluations. This includes identifying depressive episodes, distinguishing somatization symptoms, analyzing etiology, and evaluating the effectiveness of treatment programs. This review summarizes recent research on biosensing technologies for depression. Special attention is given to technologies that can be portable or wearable, with the potential to enable patient use outside of the hospital, for long periods.

## 1. Introduction

### 1.1. Depression

**Figure 1:**
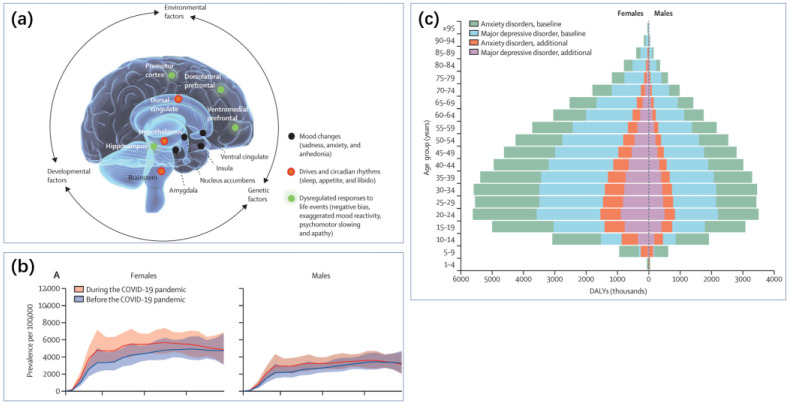
(**a**) Depression results from an interaction between multiple risk and protective factors that is unique for each person. Regardless of the distal origins of the causal pathways, they converge on brain development and function and are expressed in multiple brain regions that interact to mediate various depressive features (shown in blue, red, and green). These brain patterns are highly variable, likely reflecting etiological differences, variations in the degree of illness severity and persistence, and the heterogeneous expression of mood, motor, cognitive, and vegetative symptoms among individuals [7]. Reproduced under the terms of the Creative Commons Attribution License, Copyright 2021 by the authors, published by Elsevier Ltd. (**b**) The global prevalence of major depressive disorder (MDD) before and after adjustment for (i.e., during) the COVID-19 pandemic, 2020, by age and sex [8]. Reproduced under the terms of the Creative Commons Attribution License, Copyright 2021 by the authors, published by Elsevier Ltd. (**c**) Global burden (disability-adjusted life-years) of MDD and anxiety disorders by age and sex [8]. Reproduced under the terms of the Creative Commons Attribution License, Copyright 2021 by the authors, published by Elsevier Ltd.

Depression, as a global public health challenge, stands as a significant issue in the field of mental health (Figure 1a) [1]. According to the World Health Organization (WHO), over 350 million people worldwide are afflicted with depression, accounting for more than 4% of the global population, and this number continues to rise annually [2]. This global health crisis exerts a profound and widespread impact on human society. Annually, approximately 800,000 individuals choose to end their lives, with the disabilities and economic losses resulting from suicide (and attempted suicide) being immense [3]. Suicide has become the second leading cause of death among individuals aged 15 to 29, highlighting the ruthless toll that depression takes on the younger generation [4].

The repercussions of this illness extend beyond individual health, permeating various facets of the socio-economic fabric. In the United States alone, the loss of productivity and medical expenses due to depression amounted to a staggering USD 210.5 billion in 2018 [5]. Family members and friends also bear significant burdens, enduring additional emotional stress and caregiving responsibilities. On a societal level, decreased labor market participation and increased public health system burdens exacerbate the pressure on depression patients, creating a complex vicious cycle. During major events such as the COVID-19 pandemic, the incidence of depression rises, particularly among healthcare workers facing significant stress (Figure 1b) [6].

The potential triggers for depression include genetic predisposition [9], life stress, obesity [10], inflammation or cancer, and childbirth [11], among others. Gender and age are also influential factors in the development of depression (Figure 1c). Depression often does not manifest in isolation; it is accompanied by symptoms such as insomnia and reduced activity [12]. Disorders like insomnia can also increase the risk of depression. Depression is closely linked with various other health issues such as cardiovascular diseases, diabetes, and obesity, further compounding the physical burden on patients [13]. Consequently, the diagnosis and treatment of depression are complex and long-term processes. Longitudinal studies in primary or secondary healthcare settings show that the recurrence rate can be as high as 71–85% over five years or more [7].

### 1.2. Personalized Treatment and Biosensing

**Figure 2:**
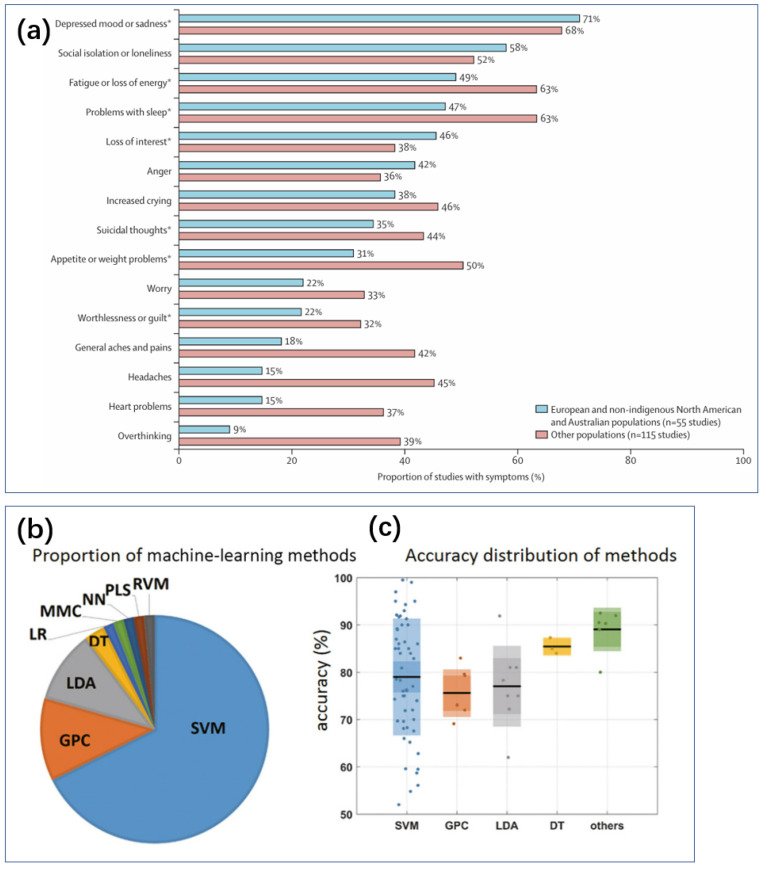
(**a**) Depressive symptoms in diverse global populations [7]. Data from Haroz and colleagues, from 170 study populations and 76 nationalities or ethnicities [19]. * ICD or DSM major depressive episode symptoms. Reproduced under the terms of the Creative Commons Attribution License, Copyright 2021 by the authors, published by Elsevier Ltd. (**b**) The number of papers on various machine learning classification methods in MDD studies, 2000–2017 [20]. (**c**) Box plots of 66 articles on MDD recognition accuracy based on five methods [20]. Reproduced with permission, Copyright 2018 John Wiley & Sons Ltd.

Personalized treatment represents a significant direction for the advancement of depression management. Given the complex etiology and manifestations of depression, the goal is to help each individual select the treatment most likely to yield positive results, thus achieving precision medicine [14]. Individual manifestations of depression are very diverse (Figure 2a). Although current depression scales can be used for self-diagnosis, their high subjectivity and poor correlation between different scales present challenges [15]. Misattributing depressive symptoms and signs to physical illnesses, and consequently failing to recognize the potential for the somatization of depression, also impacts treatment outcomes [7]. Furthermore, the efficacy of antidepressant medications varies significantly among patients. Particularly for those with mild depression, research has shown that Cohen’s effect size estimate between drug treatment and placebo groups is less than 0.2 [16].

Preventive and interventional measures for depression are preferable to bearing the substantial burden and high suicide rates once individuals progress to severe depression [2,5]. The development of machine learning has made more widespread, cost-effective, and accurate personalized and precision medicine for depression possible (Figure 2b) [17]. Analyzing depression based on internet behavior and providing personalized treatment have been proven beneficial for managing depression [18]. In this context, to more effectively provide targeted treatment and evaluate the effectiveness of existing interventions in real time, there is a need for biosensing technologies capable of continuous monitoring. This is particularly advantageous for providing low-cost assistance to a large number of potential or mildly depressed patients.

Biosensing technology, through the detection of biomarker changes, can provide objective, real-time physiological data, aiding in the more accurate identification of depression symptom patterns and underlying mechanisms. These technologies range from neuroimaging to genomics to more portable wearable devices, collectively forming a multidimensional monitoring system that allows for in-depth exploration of the biological basis of depression. By integrating the latest advancements in biology, engineering, and psychology, biosensing technology not only holds the promise of improving the early detection rates of depression but also provides critical information for designing personalized treatment plans, thereby improving patient outcomes and alleviating societal burdens.

### 1.3. Summary

This review aims to summarize the recent advancements in biosensing technologies applied to the diagnosis of depression, exploring their advantages, limitations, and future directions. The goal is to provide a comprehensive perspective for researchers and clinicians in the field, fostering interdisciplinary collaboration and promoting innovation and progress in depression diagnosis and treatment technologies. This review primarily covers the identification of depressive episodes, detection of etiological biomarkers or symptoms, and recognition of somatization phenomena.

Given the diverse and complex physiological information involved in depression, this review focuses on technologies that are portable or wearable and have the potential for long-term use by patients outside of hospital settings. The discussion is divided into two main sections: biochemical sensing of internal chemical biomarkers and wearable technologies for other physiological signals

## 2. Biochemical Sensing

Currently, laboratory tests and biomarkers play a crucial role in the diagnosis of depression, with researchers having identified numerous biochemical markers associated with the disorder. Unlike general mood disturbances, pathological depression is often accompanied by abnormalities in physiological metabolism. Many antidepressant medications are developed based on these metabolic processes related to mood and mental health. Thus, these biomarkers are important targets for biosensing in depression diagnosis. There are also strong correlations among different types of biomarkers, which can aid in systematically analyzing the etiology and condition of depression [21].

### 2.1. Hormone Sensing

In the diagnosis and detection of depression, hormone testing, though not a standard method, can reveal changes in certain hormone levels that may be associated with depression [22]. For instance, individuals with severe depression often exhibit impaired signaling in the corticosteroid receptor (CR) pathways, leading to the increased production and secretion of corticotropin-releasing hormone (CRH) in various brain regions [22]. Additionally, hormone testing can serve as an auxiliary means to help rule out other diseases that might cause mental states similar to depression. For example, elevated cortisol levels are commonly found in individuals with depression [23]. Abnormal thyroid hormones, such as hypothyroidism or hyperthyroidism, can also lead to changes in mood and mental state resembling depressive symptoms. Variations in sex hormones (such as estrogen and testosterone) can similarly influence mood, while melatonin, which is involved in sleep regulation, often shows dysregulated secretion in depressed patients, affecting their sleep and mood. Although these hormonal changes can provide valuable information, they are not standalone diagnostic indicators but can contribute to a comprehensive diagnostic assessment.

#### 2.1.1. Cortisol Sensing

Cortisol, a glucocorticoid hormone produced from cholesterol, plays a significant role in the development of depression due to potential functional defects in glucocorticoid receptors. Cortisol is also involved in the metabolism of serotonin (5-HT) and the hypothalamic–pituitary–adrenal (HPA) axis, potentially affecting depression through multiple pathways [23,24,25]. Numerous studies have reported the use of cortisol and related metabolic pathways in the treatment of depression, including the treatment of sleep disorders strongly associated with depression [23,26,27,28,29].

**Electrochemical Sensing**

As a small-molecule steroid, cortisol is often detected using receptor recognition and electrochemical detection methods, employing specific structures for selective recognition. For example, Zhang et al. developed a sensor based on ZnO nanostructures to monitor stress levels and recovery times in high-performance athletes [30]. Using electrodeposition techniques, ZnO nanorods (ZnO NRs) were deposited on a glassy carbon electrode (ZnO/GCE) surface, and 3-aminopropyltriethoxysilane (APTES) was applied to the electrode surface to enhance the immobilization and stability of cortisol antibodies (C-M ab). APTES served as a coupling agent to covalently link C-M ab with ZnO nanorods, enhancing the stability and immobilization capacity of C-M ab and preventing leaching or denaturation. The resulting device achieved a detection range of 10^−6^ nM to 10^6^ nM and a detection limit of 2 × 10^−4^ nM.

**Figure 3:**
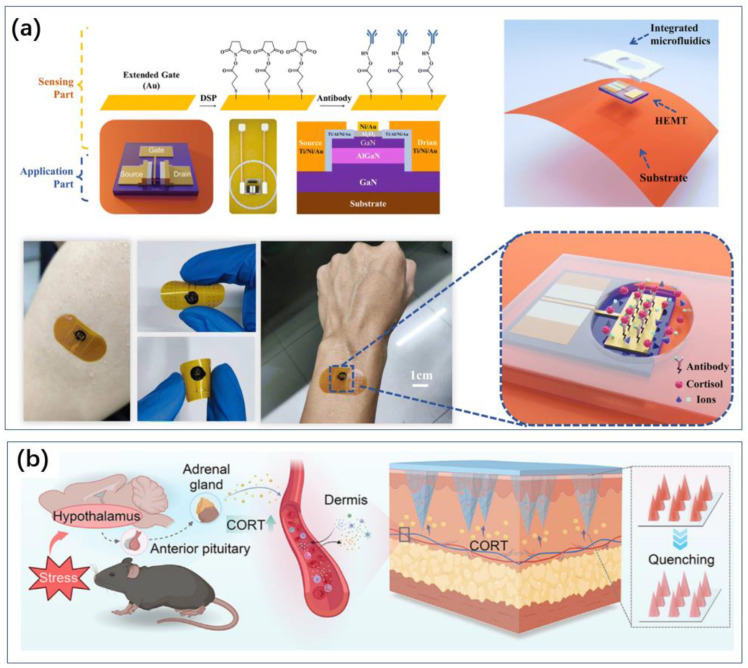
Transform devices that would otherwise be difficult to use at home into wearable sensors, which in turn enable convenient monitoring. (**a**) Flexible substrate turns field effect tube sensors into wearable sensors for cortisol sensing [32]. Reproduced with permission, Copyright 2024 ROYAL SOCIETY OF CHEMISTRY. (**b**) Microneedling allows for the detection of markers in subcutaneous capillaries, enabling wearable sensors for blood sample sensing [36]. Reproduced with permission, Copyright 2024 American Chemical Society.

Similarly, utilizing antibody-based electrochemical sensing, Sharma et al. innovated by using graphene’s exceptional conductivity and physicochemical properties [31]. They proposed a pyrene butyric acid N-hydroxysuccinimide ester (PBASE-NHS)-modified commercial graphene foam (GF) electrode for the ultrasensitive detection of cortisol in human saliva. The structure involved monoclonal anti-cortisol antibodies (mAb-cort) attached to the PBASE-NHS/GF electrode, non-covalently immobilized on the vertically aligned graphene foam electrode surface. This unique immobilization strategy preserved the structural integrity and conductivity of graphene while promoting antibody immobilization. Using differential pulse voltammetry (DPV) to detect the binding of cortisol with immobilized monoclonal antibodies, the sensor achieved a detection range of 1.0 fg/mL to 10,000 pg/mL and a detection limit of 0.24 fg/mL. The flexibility of graphene electrodes means that there is potential to develop wearable sensors. Some of the sensors can also realize wearable cortisol sensing by preparing flexible substrates [32] (Figure 3a).

Moreover, extensive research has introduced innovations and changes in the selection of receptors, such as the increasingly common use of molecularly imprinted polymers (MIPs). Using the target molecule as an imprint template allows the sensor to obtain complementary binding sites for the analyte, thereby eliminating the need for additional labeling procedures and external probes to recognize and bind the target [33,34]. When the analyte binds to the imprint cavities, electron transfer is impeded, allowing for the determination of analyte concentration through changes in redox current [35].

**Figure 4:**
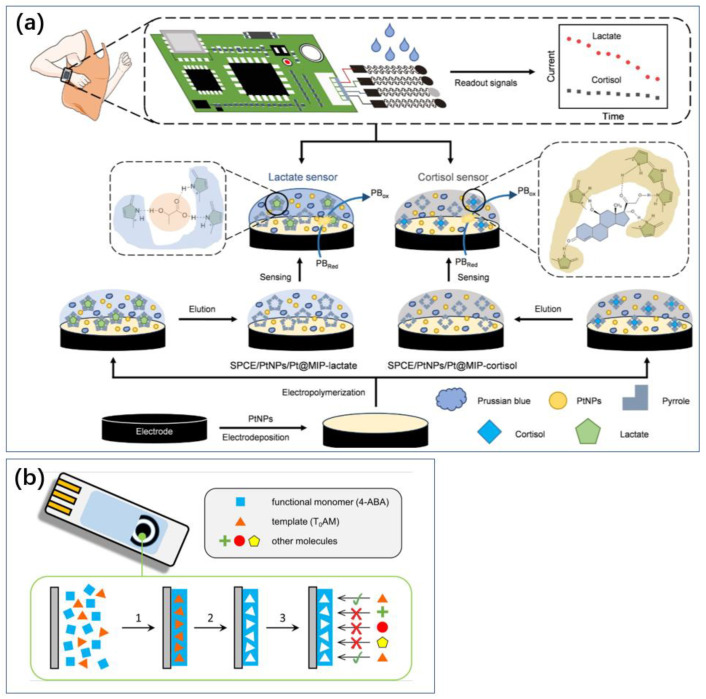
MIP method for sensing without consumables. It can be used to develop wearable or portable sensors. (**a**) Wearable technology to detect cortisol in sweat using MIP technology [37]. Reproduced with permission, Copyright 2024 Wiley-VCH (**b**), and a miniaturized sensor for thyrotropic protamine [38]. Reproduced with permission, Copyright 2019 Elsevier.

For instance, Pei et al. developed a flexible MIP sensor using cortisol or lactate as template molecules, pyrrole (Py) as the functional monomer, and incorporating platinum nanoparticles (PtNPs) to enhance electron transfer capabilities (Figure 4a) [37]. This sensor is used for the real-time monitoring of cortisol and lactate levels in sweat. Prussian Blue (PB) was embedded within the MIP as an internal redox probe, eliminating the need for additional probes and facilitating the simultaneous quantification of cortisol and lactate concentrations, thereby enhancing sensor sensitivity. The flexible cortisol and lactate MIP sensor achieved a low limit of detection (LOD) of 1.07 nM and 1.09 mM, respectively, and high sensitivity (0.09 μA lg [nM]^−1^ and 1.28 μA lg [nM]^−1^, respectively), and it exhibited excellent stability and selectivity. This flexible MIP sensor can continuously monitor changes in cortisol and lactate concentrations in sweat and can be integrated into wearable devices for everyday use.

Mani and Anirudhan reported a simple electrochemical biosensor for cortisol (Cor) detection using MIPs [39]. They prepared the MIP structure by grafting allyl gold nanoparticles and carboxylated graphene oxide (Au/GO-COOH) with the template molecule Cor using copolymerization. Electrochemical impedance spectroscopy and cyclic voltammetry confirmed that the sensor exhibited good electrocatalytic activity toward Cor, with the nanomaterials and electroactive sites on the MIP collectively enhancing electron transfer rates. Differential pulse voltammetry revealed a detection range of 1 × 10^−3^ M to 1 × 10^−14^ M and a detection limit of 0.61 × 10^−14^ M.

**Optical Sensing**

Research on optical sensors for cortisol is also prevalent. Common optical biosensing techniques include surface plasmon resonance (SPR) [40,41] and fluorescence methods [42,43,44]. Liu et al. developed an S-flex fiber optic (SFFO) sensor based on localized surface plasmon resonance (LSPR) for the quantitative measurement of cortisol [45]. The SFFO structure allows for the generation of an efficient evanescent field to excite LSPR phenomena on noble metal nanoparticles (NPs) on the probe surface, with MoS2-NPs modifying the sensor to enhance the performance of the cortisol antibody sensing probe. The sensor achieved a sensitivity of 3.07 nm/log (ng/mL) and a detection limit of 148.5 pg/mL.

**Figure 5:**
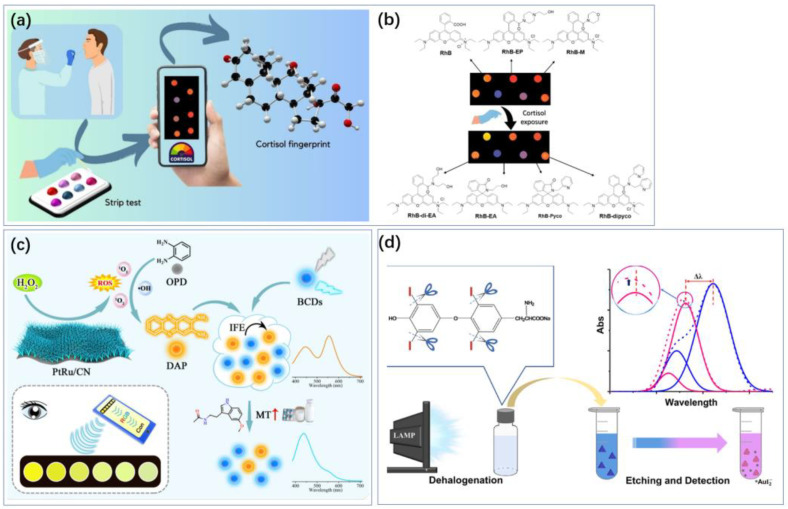
Colorimetry makes it possible to achieve sensing using smartphones and even the human eye. (**a**) Cortisol sensing using a smartphone camera. (**b**) Real images of the strip test under a UV lamp at 365 nm before and after cortisol exposure [47]. Reproduced with permission, Copyright 2024 WILEY—V C H VERLAG GMBH & CO. KGAA (**c**) Colorimetric sensing of melatonin was achieved using blue-emissive carbon dots (BCDs) [48]. Reproduced under the terms of the Creative Commons Attribution License, Copyright 2024 by the authors, published by Elsevier B.V. (**d**) The morphological changes of AuTNPs resulted in vivid color variations of the nanoprism dispersion, accompanied by a blue shift of the in-plane LSPR peak, enabling visual and photometric sensing [49]. The blue and red dotted lines are the original extinction spectra obtained from intact and etched AuTNPs, respectively. Reproduced with permission, Copyright 2019 American Chemical Society.

Optical methods can manifest as changes in color or luminous intensity, which makes sensing possible using portable devices such as smartphones or even the human eye (Figure 5). And there is no need for electrochemical methods of sensing circuits to power the electrodes. Liu et al. also established a sensitive method using a wearable Eu-MOF microneedle patch to simultaneously detect cortisol through visible fluorescence quenching [46]. The europium metal–organic framework (Eu-MOF) embedded in the matrix played a crucial role in cortisol recognition and quantitative analysis. The strong interaction between cortisol and Eu-MOF enabled effective quantitative analysis through fluorescence quenching, with high sensitivity, a detection range of 10^−7^ to 10^−3^ M, and a detection limit as low as 10^−9^ M. Santonocito et al. designed novel fluorescent probes that interact with non-covalent interactions with cortisol. Sensing can be realized via smartphones [47] (Figure 5a,b).

#### 2.1.2. Thyroid Hormone Sensing

Thyroid hormones, secreted by the thyroid gland, play critical roles in promoting nutrient metabolism, brain development, and the proper functioning of the nervous and cardiovascular systems [50,51]. Numerous clinical reports have indicated that thyroid hormones, particularly triiodothyronine (T3) and L-thyroxine, can influence mood and have been proven effective in treating depression [52,53,54,55]. Current sensing methods primarily focus on electrochemical and optical sensing techniques.

**Electrochemical Sensing**

Mradula et al. developed a label-free electrochemical immunosensor for thyroxine detection by immobilizing a thyroxine-specific monoclonal antibody (Ab) on a composite of the copper metal–organic framework (Cu-MOF) and polyaniline (PANI)-modified screen-printed carbon electrode (SPCE) [56]. The concentration of thyroxine was measured using cyclic voltammetry (CV) and DPV. The sensor exhibited a dynamic linear range of 10–10^5^ pM, with a low LOD of 0.33 pM (0.25 pg/mL) for CV and 0.17 pM (0.13 pg/mL) for DPV, demonstrating good recovery and repeatability.

Park et al. designed a DNA three-way junction (3WJ) structure as a multifunctional bio probe, capable of target detection, electrochemical signal reporting, and immobilization [57]. The multifunctional DNA was fixed on a porous rhodium nanoplate (pRhNPs)-hybrid-modified gold micro-gap electrode. pRhNPs increased the surface area and facilitated signal amplification. Thyroxine (T4) detection was performed using CV and electrochemical impedance spectroscopy (EIS), with a detection limit of 10.33 pM under optimal conditions. Clinical samples could detect T4 concentrations as high as 11.41 pM.

Singh et al. developed molecularly imprinted polyaniline by creating specific geometric cavities for thyroxine through the removal of the hormone from the polyaniline matrix [58]. The imprinted polymer was introduced onto an indium tin oxide (ITO)-coated glass electrode. Thyroxine concentration was measured using CV, with a detection range of 5–50 pg/mL and a detection limit of 6.16 pg/mL. This method was found to be more sensitive than other reported techniques and showed good recovery in saliva, making it a useful non-invasive diagnostic tool for daily use.

Pacheco et al. also realized an MIP electrochemical sensor for p-thyroxine amine (T_0_AM) on the surface of screen-printed carbon electrodes, with 4-aminobenzoic acid (4-ABA) as the building and functional monomer and the analyte T_0_AM as the template (Figure 4b) [38]. MIP methods are often used for wearable or portable sensors because they enable label-free sensing and are easy to store without active substances (Figure 4).

**Optical Sensing**

Borah et al. established a cost-effective colorimetric method for detecting ascorbic acid (AA) and thyroxine (TH) by synthesizing silver nanoparticles (AgNPs) modified with epigallocatechin gallate (EGCG) and cetyltrimethylammonium bromide (CTAB) [59]. Changes in the LSPR properties of AgNPs were used as the standard for AA and TH detection. The detection ranges for AA and TH were 0.1–0.7 mM and 0.1–0.4 mM, respectively, with detection limits of 0.67 mM and 0.33 mM. This method is characterized by its simplicity, eco-friendliness, cost-effectiveness, and time efficiency.

Using dispersive photometry, Ren et al. detected iodide (I−) and L-thyroxine by etching gold triangular nanoplates (AuTNPs) in the presence of H_2_O_2_ [49]. The morphological changes in AuTNPs caused significant color changes in the nanoprisms, accompanied by a blue shift in the in-plane LSPR peak, enabling visual and photometric sensing (Figure 4d).

#### 2.1.3. Sex Hormone Sensing

Sex hormones, primarily estrogen and testosterone, are vital steroids secreted by the gonads of female and male individuals, respectively. Sex hormone levels affect the onset of depression and anxiety, including by affecting structures in the brain [60] and interacting with their markers of depression [61], which also contributes to gender differences in depression [62]. Therefore, real-time, efficient, and responsive detection of these hormones is crucial.

**Testosterone Sensing**

Velayutham et al. developed a sensor for testosterone detection by immobilizing specially designed DNA onto a screen-printed gold electrode (SPGE) modified with a composite of conductive hydrogel and gold nanoparticles (HG/NP) [63]. The incorporation of HG/NP not only enhanced the sensor’s conductivity but also acted as an antifouling layer, minimizing signal interference from nonspecific biomolecular interactions in complex biological samples such as human serum. The sensor was evaluated using CV, electrochemical impedance spectroscopy (EIS), and square-wave voltammetry (SWV). It exhibited a detection range of 0.05 to 50 ng/mL, with a detection limit of 0.14 ng/mL and a sensitivity of 0.23 μA ng⁻^1^ mL cm⁻^2^, demonstrating good selectivity.

Sanchez-Almirola et al. reported an electrochemical sensing platform based on MIPs [64]. They prepared MIPs by electropolymerizing o-phenylenediamine (oPD) on micro-screen-printed carbon electrodes (SPCEs) using CV, resulting in poly-o-phenylenediamine (PoPD). The MIP-PoPD/SPCE was used for direct electrochemical sensing of testosterone at low physiological levels. The sensor achieved an LOD of 1 ng/dL and a detection range of 1 to 25 ng/dL. This sensing chip can interface with mobile devices and be operated via a smartphone, catering to personalized health needs. The inherent elasticity and flexibility of MIPs make them suitable for technologies involving wearable sensors.

**Estrogen Sensing**

In addition to testosterone, estrogen detection is also essential for diagnosing and managing various health conditions. Research has been conducted to develop sensitive and selective estrogen sensors.

Tortolini et al. developed a simple and sensitive sensor for 17-β-estradiol (E2) by functionalizing graphite screen-printed electrodes (GSPEs) through a two-step method: (1) drop-casting and depositing gold nanoparticles (AuNPs) and (2) electropolymerized methylene blue (MB) [65]. The analyte was detected using cyclic voltammetry, and the synergistic effect of AuNPs and PMB resulted in a wide linear range of 0.5 to 125.0 μmol/L, with an LOD of 41 μmol/L. The sensor exhibited long-term stability, good reproducibility (RSD = 2.9% for *n* = 10), and selectivity.

Integrating electrochemical and optical sensing properties, Cao et al. prepared a Ru(bpy)_3_^2+^/MWCNTs/Nafion/gold electrode using surface electrostatic adsorption and ion exchange, and an MIP with E1 molecular recognition capability using the sol–gel method [66]. The electrode, modified with MIP, formed an electrochemiluminescent sensor (MIP-ECL). This approach combined the high sensitivity of ECL with the high selectivity of MIP. Additionally, the incorporation of carboxylated multi-walled carbon nanotubes (MWCNT-COOH) enhanced the functionalization of the gold electrode surface, increasing the binding sites for MIP. The good conductivity of MWCNTs facilitated electron transfer, further improving the sensor’s sensitivity. The sensor demonstrated a detection range of 0.1 to 200 μg/L with an LOD of 0.0047 μg/L.

#### 2.1.4. Melatonin Sensing

Melatonin is an indoleamine hormone produced by the pineal gland under dark conditions and plays a critical role in regulating circadian rhythms, memory, dreaming, and other functions [67,68]. The accurate and efficient detection of melatonin is essential for monitoring depression and understanding its relationship with sleep and the light environment [69,70]. For some depression patients, melatonin and drugs targeting its metabolic pathways can be effective treatments [71,72,73]. Current research primarily focuses on electrochemical and optical sensing methods.

**Electrochemical Sensing**

Richard et al. developed a sensing platform by combining Nb_2_CTx MXene nanosheets with zinc-based metal–organic frameworks (ZnMOFs) [74]. The ZnMOF was synthesized using zinc ions as the metal component and L-glutamic acid as the organic linker. This novel composite material (Zn-MOF-Nb_2_CTx MXene) exhibited enhanced electrocatalytic performance, improved conductivity, and increased active sites, making it suitable for the electrochemical detection of melatonin (MEL). The sensor, made from carbon yarn (CY) coated with Zn-MOF-Nb_2_CTx MXene nanocomposite, demonstrated a linear detection range for MEL of 1 to 100 μM with a detection limit of 215 nM, showing high selectivity. Notably, the synergy between the MOF nanosheets and MXene nanosheets significantly improved the electrochemical performance for MEL detection. Additionally, the ZnMOF-Nb_2_CTx-MXene nanocomposite-coated CY could be integrated into commercial adhesive bandages, creating a prototype device with a detection limit of 349 nM, indicating its potential for wearable medical applications.

**Optical Sensing**

Kumar et al. used a 3,6-diaminocarbazole (DAC) fluorescent probe for melatonin (MLT) detection, offering high sensitivity, selectivity, and simplicity [75]. The study creatively utilized a ratiometric fluorescence technique based on the inner filter effect (IFE). Upon adding MLT, the photoluminescence of DAC exhibited a strong quenching response at 448 nm, while a new emission at 343 nm emerged from the DAC-MLT interaction, increasing with MLT concentration. These signal changes were used for ratiometric fluorescence detection of MLT. A linear relationship was observed between the emission intensity ratio and MLT concentration within a range of 0 to 78 μM, with a detection limit of 30 nM. Additionally, a smartphone application “RGB Color Detector” was demonstrated to be very useful for detecting color changes. Wang implemented colorimetric sensing of melatonin using blue-emitting carbon dots (BCDs), which can be measured with the naked eye and RGB sensors, with an LOD of 23.56 nmol/L (Figure 4c) [48].

**Photoelectrochemical Sensing**

Sun et al. combined electrochemical and optical sensing technologies to develop a photoelectrochemical (PEC) sensor for melatonin using graphene oxide nanoribbons (GONRs) synthesized through a microwave-assisted method. GONRs served as electrocatalysts on screen-printed carbon electrodes (SPCEs) to facilitate melatonin detection [76]. The PEC evaluation utilized light-emitting diodes (LEDs) and a solar simulator as light sources. CV indicated that the Faradaic current for melatonin oxidation was amplified on GONR-modified SPCEs under LED and simulated sunlight illumination, with a detection range of 100 μM.

#### 2.1.5. Partial Summary in Hormone Sensing

Electrochemical sensing against hormones is mainly achieved by means of antibodies, functionalized DNA, and MIP, and carbon materials such as graphene and metal nanoparticles are used to amplify signals or immobilize substrates. For melatonin, there are organic small-molecule electrocatalytic-type electrodes.

The advantage of hormone sensing is that such markers are secreted by cells into the humoral environment and carried by the circulatory system throughout the body. Therefore, good sensing of hormones can be achieved based on most collected blood samples and even many non-invasive samples such as urine, sweat, and saliva. The fact that it can be collected painlessly and safely makes it ideal for home use. But hormones, for the most part, do not directly correlate with depression or neurological activity, and achieving a diagnosis of depression based on hormones alone is difficult. Hormonal biosensing can be utilized to guide the treatment of patients who have a diagnosed cause or whose disease manifestations are metabolically related to the disease.

### 2.2. Cytokine Sensing

Cytokines are low-molecular-weight proteins secreted by immune and some non-immune cells in the human body, playing regulatory roles in various physiological and metabolic activities, including immunity, verification, and cancer [77]. Studies have shown that MDD is accompanied by immune dysregulation and the activation of the inflammatory response system (IRS) [78]. This often manifests as abnormal cytokine concentrations, such as elevated levels of interleukin-6 (IL-6) and tumor necrosis factor-α (TNF-α), in patients with depression. Statistical analyses indicate a positive correlation between depression and IL-1 and IL-6, with body mass index (BMI) potentially serving as a mediating/modulating factor [79]. Cytokines like IL-6 and TNF-α are involved in inflammation and immune responses, and their levels are frequently elevated in depressed patients, further supporting the link between inflammation and depression. Depressed patients with increased inflammatory biomarkers are more likely to exhibit treatment resistance, and, in some studies, antidepressant treatment has been associated with a reduction in inflammatory responses [80]. Immune signals from the immune system to the brain may contribute to the onset or exacerbation of depression and other diseases, but the intracellular molecular mechanisms underlying the inflammation–depression connection remain to be elucidated [21,81].

#### 2.2.1. Interleukin Sensing

Interleukins (ILs) are a class of pro-inflammatory cytokines, specifically lymphokines, that mediate interactions between leukocytes. These interleukins encompass a variety of cytokines that play crucial roles in activating and regulating immune cells, mediating T- and B-cell activation, proliferation, differentiation, and inflammatory responses [82,83,84,85]. According to a meta-analysis summarizing decades of research, using a random-effects model analysis, the correlations of IL-1 and IL-6 with depression were found to be higher than that of CPR [79]. Recent studies have also reported correlations between other cytokines and depression, suggesting the potential development of depression-relieving drugs based on these findings [86,87,88,89]. Various types of ILs are associated with different disease-related metabolic or immune responses, making them useful in diagnosing depression in specific populations. For instance, IL-8 has shown correlations with depression in breast cancer patients [90], IL-17A in postpartum women [91], and IL-23 in psoriasis patients [92]. Accordingly, specific medications can be selected based on the patient’s condition; for example, IL-1β is related to myocardial infarction, and downregulating its concentration with the demethylase Jmjd3 can improve post-infarction depression [93].

Research on IL-6 is the most extensive due to its higher significance in depression [78]. Electrochemical sensing has also been realized in the detection of ILs. Buckey et al. developed an electrochemical immunoassay for IL-6 by capturing IL-6 with magnetic beads and generating electrochemical signals using horseradish peroxidase/tetramethylbenzidine [94]. This method achieved IL-6 detection in a range of 50–1000 pg/mL, relevant to the physiological range in various biological systems. Similarly, Narayanan et al. employed electrochemical sensing using antibody probes, depositing a conductive copper (Cu) layer on a zinc oxide (ZnO) film on graphite sheets (GSs) [95]. The conductive Cu layer glazing on the ZnO film led to enhanced sensing behavior, with a detection limit as low as 0.43 pg/mL. Ghosh et al. also realized electrochemical sensing of IL-6 using electrodes made from graphene conductive ink [96]. This device exhibited good flexibility, making it an ideal choice for wearable and stretchable bioelectronics applications with the potential for wearable biosensing.

Zhang et al. achieved non-invasive IL-6 detection, beneficial for long-term depression patients by avoiding the pain of frequent blood sampling [97]. They developed an ultra-sensitive electrochemical immunosensor for quantitative detection of IL-6 in exhaled breath condensate (EBC) using boron nitride nanosheet/gold nanoparticle (BNNS/AuNP) hybrids. The two-dimensional morphology and large surface area of BNNS facilitated enhanced antibody loading, while the high conductivity of AuNPs accelerated electron transfer, amplifying the electrochemical signal. BNNS was synthesized via chemical vapor deposition and modified with AuNPs, deposited on screen-printed carbon electrodes, and anti-IL-6 antibodies were immobilized through EDC/NHS cross-linking. The immunosensor used differential pulse voltammetry to detect IL-6 in a linear range of 0.01–200 ng/mL, with a detection limit of 5 pg/mL.

Ting et al. utilized MIP for IL-6 sensing [98]. They developed a highly sensitive and selective IL-6 sensing platform by depositing P(o-PD)-based MIP on oxygen-functionalized screen-printed carbon electrodes containing gold nanoparticles, 3-aminopropyltriethoxysilane (APTES), and glutaraldehyde (GA). The adsorption of redox probes on APTES and the enhanced conductivity of the protein surface by gold nanoparticles accelerated electron transfer on the electrode surface, increasing peak current. The functionalized surface improved hydrophilicity due to the presence of amino and carbonyl groups. The IL-6 detection concentration range by DPV was 2–400 pg/mL, with a sensitivity of 3.48 μA/log(pg/mL) and a detection limit of 1.74 pg/mL.

Fluorescence sensing technology can also be utilized for the detection of interleukins (ILs). Zhao et al. employed quantum dots (QDs) and antibody probes to detect IL-6 [99]. Antibodies were immobilized on a polydimethylsiloxane (PDMS) array through 4-(N-maleimidomethyl)cyclohexane-1-carboxylic acid 3-sulfo-N-hydroxysuccinimide ester sodium salt (sulfo-SMCC) coupling, enhancing antigen–antibody binding efficiency. Additionally, QDs provided a fivefold increase in fluorescence intensity, enabling sensing with as little as approximately 20 μL of sample. Gaikwad et al. used single-walled carbon nanotubes (SWCNTs) to create probes, selecting L-lysine for passivation to prevent interference from other protein molecules [100]. This approach reduced the detection limit by three orders of magnitude compared to previous antibody-coupled SWCNT sensors. Ryan et al. designed a fluorescence sensor using SWCNTs with specific DNA aptamers as probes, achieving IL-6 detection with good biocompatibility [101].

Yamaguchi et al. developed an IL sensing platform based on photoelectrochemistry (PEC) [102]. They designed an IL-6 immunosensor platform using a unique three-dimensional microfluidic structure fabricated by femtosecond pulse laser processing. The 3D design facilitated the exploration of a compact biosensing system, though the need for a centrifuge hindered overall miniaturization and convenience. Zou et al. proposed a novel PEC sensor platform using optical fibers (OFs) as the working electrode to guide in situ light [103]. By introducing energy transfer between Au NPs@dsDNA and CdS quantum dots, the resulting photoelectrode exhibited nearly zero background, enabling the detection of trace amounts of IL-6 with an LOD of 0.19 pg/mL. This approach offers the potential for biosensing in extremely small fluid samples for depression research.

#### 2.2.2. Tumor Necrosis Factor Sensing

Tumor necrosis factor (TNF) is a substance capable of inducing hemorrhagic necrosis in various tumors. TNF-α is produced by macrophages, while TNF-β is produced by lymphocytes [104,105]. Studies have shown elevated levels of TNF-α receptors in patients with Type D personality and MDD, indicating a possible link between TNF and depression [106,107]. In animal models, TNF-α can induce depressive symptoms [108]. Patients with elevated plasma TNF-α levels often exhibit an association between antidepressant treatment and decreased TNF-α levels [109,110]. Blocking TNF-α has been shown to alleviate depressive symptoms. Thus, sensing TNF can be used for diagnosing and guiding the treatment of depression, especially for patients who have undergone cancer therapy. For TNF detection, we focus on both electrochemical and optical sensing technologies.

**Electrochemical Sensing**

Ondevilla et al. proposed a point-of-care (POC) electrochemical biosensor utilizing aptamer-based sensing technology [111]. To meet POC testing requirements, they combined an electrokinetic technique known as DC-biased alternating-current electrokinetics (DC-ACEK) with the proposed electrochemical sensor. This combination facilitated the rapid collection of target molecules on the aptamer-modified electrode, reducing detection time and increasing sensitivity to the picogram level. Compared to the traditional enzyme-linked immunosorbent assay (ELISA) with a detection time of 4 h, the entire operation was completed within 5 min. The LOD calculated by CV and electrochemical impedance spectroscopy (EIS) was 0.84 pg/mL, with detection ranges of 1–100,000 pg/mL.

**Optical Sensing**

Khachornsakkul et al. developed a distance-based paper analytical device (dPAD) combining MIPs and carbon dots (CDs) [112]. The dPAD utilized fluorescent CDs and MIP technology, offering high selectivity and sensitivity. Detection was based on the fluorescence quenching of CDs through interactions between the target analytes and the MIP layer on the paper substrate. This allowed for the simultaneous quantification of cytokine biomarkers such as C-reactive protein, TNF-α, and interleukin-6 (IL-6) in human biological samples. The linear ranges for these biomarkers were 2.50–24.0 pg/mL (R^2^ = 0.9974), 0.25–3.20 pg/mL (R^2^ = 0.9985), and 1.50–16.0 pg/mL (R^2^ = 0.9966), with detection limits of 2.50, 0.25, and 1.50 pg/mL, respectively. MIP technology offered cost and scalability advantages over aptamer antibodies, with a total detection time of 40 min, demonstrating considerable competitiveness.

**Figure 6:**
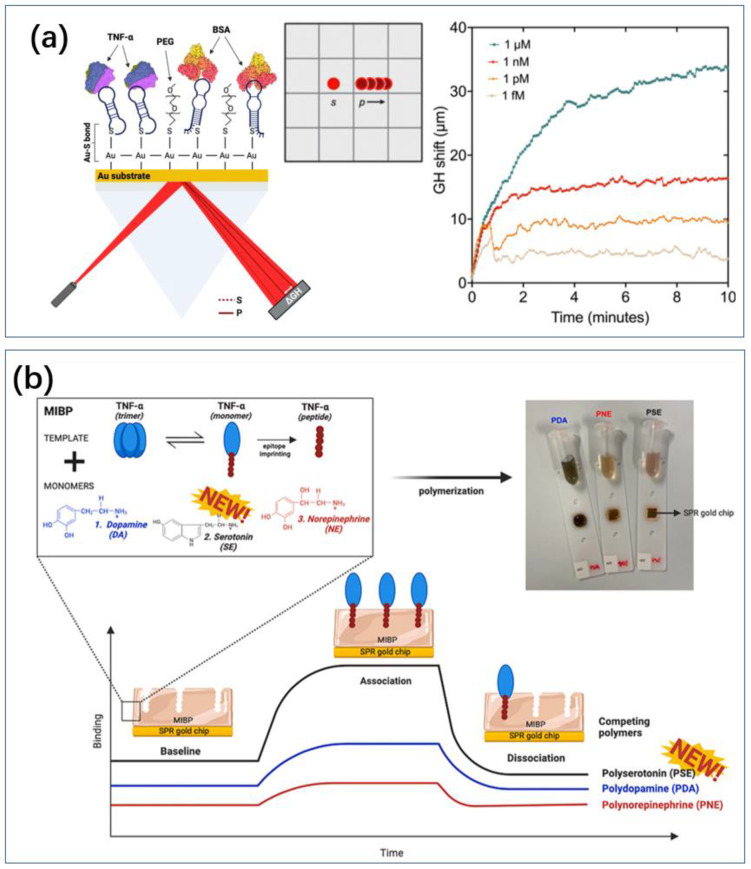
SPR sensor. (**a**) The SPR effect on the gold surface produces Goos–Hanchen uniqueness in the presence of markers, which in turn enables sensing of TNF [113]. The curves in the figure are superimposed sensorgram of titrated amount of TNF-α (1 fM to 1 μM) over a time course of 10 minutes. Reproduced under the terms of the Creative Commons Attribution License, Copyright 2023 by the authors, published by Elsevier B.V. (**b**) TNF with aptamer is attracted to the surface of gold nanolayer for SPR sensing [114]. Reproduced with permission, Copyright 2024 Analyst.

Borg et al. developed a biosensing platform in which aptamers were surface-immobilized as recognition units (Figure 6a) [113]. They analyzed the binding of targets to aptamers using the Goos–Hänchen (GH) shift at the resonance angle. This platform utilized surface plasmon resonance (SPR) substrates functionalized with aptamers and a highly sensitive GH shift measurement method for femtomolar-level detection of TNF-α, with a detection range of 1 aM to 1 μM. The GH shift increased with the amount of TNF-α. Immunoassays showed higher sensitivity, with elevated GH shifts observed at 1 fM TNF-α, potentially due to higher affinity between antibodies and TNF-α compared to aptamers. This resulted in a larger percentage of cytokine binding to surface-immobilized antibodies, leading to greater GH lateral displacement. However, aptamer-based detection offered advantages in stability, cost-effective mass production, and minimizing batch-to-batch variation compared to antibody-based detection. Batta et al. combined MIP and SPR, which allowed for label-free TNF-a sensing compared to immunoassays (Figure 6b) [114].

### 2.3. C-Reactive Protein Sensing

**Figure 7:**
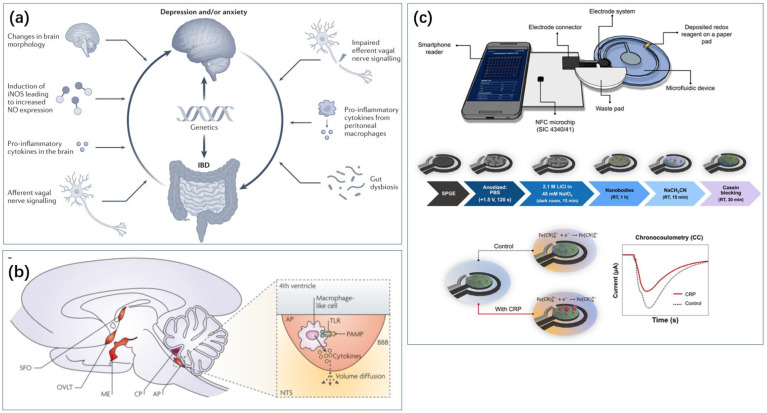
Inflammation-related sensing has important implications for the monitoring of depression. (**a**) The effect of intestinal inflammation and depression or anxiety [115]. Reproduced with permission, Copyright 2022 Springer Nature Limited. (**b**) Inflammation-related signaling pathways in the human brain [81]. Reproduced with permission, Copyright 2022 Springer Nature Limited. (**c**) Real-time monitoring of CRP with the help of cell phone NFC technology [121]. Reproduced under the terms of the Creative Commons Attribution License, Copyright 2024 by the authors, published by American Chemical Society.

There is a strong statistical association between inflammation and depression (Figure 7a) [21,79,80,81,115]. In addition to the immune-related cytokines discussed in Section 2.2, C-reactive protein (CRP) is another common biomarker. CRP is an acute-phase protein synthesized by the liver, typically elevated during inflammation or infection. Numerous studies over the past 20 years have indicated that chronic low-grade inflammation may be associated with depression, with elevated CRP levels often observed in patients with depression [116,117,118,119,120]. This suggests that inflammatory responses play a role in the pathogenesis of depression, although the underlying mechanisms remain to be fully elucidated. Particularly, studies have shown slightly differing statistical patterns, such as varying correlations between CRP and depression across different genders, with some studies reporting contradictory findings [117,120]. Nevertheless, CRP remains an important target for monitoring depression.

Gao et al. developed a label-free functionalized aptamer sensor integrated with a gold nanoparticle and carboxylated graphene oxide (AuNPs/GO-COOH) electrode for sensitive CRP detection [122]. Gold nanoparticles offer excellent stability, high conductivity, and biocompatibility, while carboxylated graphene oxide enhances the anchoring of the target molecules, improving detection accuracy. DPV achieved a wide linear range from 0.001 ng/mL to 100 ng/mL with a detection limit of 0.001 ng/mL. Wang et al. constructed a novel electrochemical aptamer biosensor using Ti3C2Tx MXene and in situ reduced Au NPs [123]. Fc(COOH) was used as a signal probe for the immobilization of thiolated RNA aptamers and CRP protein detection. The high surface area of Ti3C2Tx MXene and the high conductivity of Au NPs provided a wide linear range of 0.05 to 80.0 ng/mL for CRP with good sensitivity, as determined by DPV. Whitehouse et al. reported a DNA aptamer-based electrochemical biosensor capable of single-step and reagent-free CRP detection within 1 min, offering the potential for rapid and straightforward screening [124]. This sensor utilized methylene blue-labeled redox probes with CRP-targeting DNA aptamers, functionalized on inexpensive commercial screen-printed electrodes.

According to our current investigation, the most sensitive sensor reported was by Lin et al. [125]. They introduced a novel sandwich immunoassay method that combines surface-enhanced Raman scattering (SERS) with magnetic plasmonic nanoparticles (MPNs) to enhance sensitivity. The unique magnetic properties of these nanoparticles, combined with an external magnetic field, further increased the detection sensitivity of the SERS biosensor. Additionally, a simple statistical method called “Gaussian fractal” was introduced, which involves fractal analysis of two-dimensional Raman mapping data followed by Gaussian fitting. This method reduces the inherent variability in Raman signal measurements, making data interpretation more consistent and reliable. Using this approach, a biosensor targeting CRP achieved a detection limit of 5.96 fg/mL, which was further improved by 5.7-fold to 1.05 fg/mL under a 3700 G magnetic field.

In terms of portable diagnostics, relevant research has also been reported. Boonkaew et al. proposed a smartphone-controlled NFC potentiostat integrated with a continuous-flow microfluidic device for capturing and quantifying CRP (Figure 7c) [121]. The electrochemical analysis was conducted using a three-electrode system consisting of a working electrode (WE, 3 mm diameter), a counter electrode (CE), and a reference electrode (RE). Anti-CRP nanobodies were covalently anchored to the WE, achieving a detection range of 0.01 to 100 μg/mL with a detection limit of 1.18 ng/mL.

### 2.4. Neurotrophic Factor Sensing

Another important class of biosensing biomarkers is neurotrophic factors (NTFs). These are protein molecules produced by tissues innervated by neurons (such as muscle) and astrocytes, essential for neuron growth and survival, and closely related to neural signaling pathways and physiological regulation [126]. NTF levels are significantly associated with various neuropsychiatric disorders, including depression [127,128]. Given their small molecular weight, NTF biosensing shares similarities with interleukin (IL) detection.

One key NTF is brain-derived neurotrophic factor (BDNF), which plays a critical role in the pathophysiology of depression [129]. BDNF is widely present in the central nervous system and is crucial for neuronal survival, growth, and plasticity. Serum or plasma levels of BDNF are typically lower in patients with depression, and antidepressant treatments can increase BDNF levels, suggesting its central role in depression pathology [130,131]. The mechanisms of action of many antidepressant drugs are also linked to BDNF [129]. Although biosensing research on BDNF is less extensive compared to CRP or ILs, it encompasses various methods, including electrochemical and molecularly imprinted techniques.

**Antibody-Based Electrochemical Sensing**

Akhtar et al. designed a dual-probe immunosensor (DPI) for detecting BDNF in the extracellular matrix of neuronal cells. One probe serves as the working probe, while the other functions as a bioconjugate-loaded probe. AuNPs were used to immobilize BDNF antibodies, achieving a linear range of 4.0 to 600.0 pg/mL with a detection limit of 1.5 pg/mL [132]. Wei et al. improved the probe by modifying indium tin oxide-coated polyethylene terephthalate (ITO-PET) with nitrogen-doped graphene-polyaniline (NG-PANI) and AuNPs to enhance conductivity and protein loading capacity, achieving an LOD of 0.261 pg/mL [133]. Yoo et al. reported a BDNF detection limit of 100 fg/mL using anti-BDNF antibodies coated on a poly(dimethylsiloxane) (PDMS)-based microfluidic channel chip [134].

**Molecularly Imprinted Polymers**

Kidakova achieved BDNF sensing based on MIPs for the first time [135]. The BDNF-MIP/SPE electrochemical sensor, created through controlled/radical photopolymerization on screen-printed electrodes (SPEs), could detect BDNF as low as 6 pg/mL, even in the presence of interfering human serum albumin (HSA) protein. Ayankojo et al. developed a surface-imprinted sensor using thin-film metal electrodes, achieving a detection limit of 9 pg/mL for BDNF, with selectivity for closely related neurotrophic factor proteins (CDNF and MANF) exceeding five-fold and for proteins with similar isoelectric points (e.g., CD48) exceeding thirty-fold [136].

**Specialized Research**

Li et al. focused on BDNF target sequences, installing capture probes on aluminum micro-comb electrodes on silicon chips for the selective detection of BDNF gene sequences [137]. This approach could aid in developing suitable treatments for BDNF-related disorders. Lastly, Elfving et al. developed an ELISE kit for BDNF detection in rat blood [138], while Mandel et al. used ELISE to detect BDNF in human saliva samples [139], indicating the potential for non-invasive fluid sample kits targeting BDNF in the future. Chowdhury et al. investigated DNA aptamers for specific recognition of BDNF, identifying highly sensitive and selective aptamers [140]. Nakajima et al. designed fluorescent indicators targeting BDNF to visualize endogenous BDNF secretion from hippocampal neurons [141]. However, to date, no biosensors based on such probes have been reported, highlighting the significant development potential in the field of depression biosensing.

### 2.5. Partial Summary in Biochemical Sensing

Section 2.2, Section 2.3 and Section 2.4 describe a variety of protein macromolecular markers. Each of these markers is strongly associated with the development and treatment of a particular type of depression and is important for the patients concerned.

Electrochemical sensing targeting proteins is mainly realized by means of antibodies, aptamers, and MIPs. In addition to the signal amplification methods that have appeared before, sandwich methods and further immobilization of fluorescent substances on probes are reported due to the potentially small amounts of protein substances. Also, materials such as carbon nanotubes have nonspecific adsorption to macromolecules, so many studies have avoided interference via methods such as surface modification.

Since the amount of protein material in the sample tends to be small, achieving good signal amplification without consumables in wearable or portable devices is the key for the related technologies to be further applied.

### 2.6. Neurotransmitter Sensing

Neurotransmitters are chemical substances that transmit information between neurons or between neurons and effector cells such as muscle cells and gland cells. Due to their critical role in neural signaling, neurotransmitters are of significant importance in the diagnosis and treatment of depression [142,143,144,145]. Many antidepressant treatments target neurotransmitters or their receptors. Levels of neurotransmitters and their receptors can be used to describe the degree of stress and the efficacy of antidepressant treatments. For instance, prolonged antidepressant therapy can lead to sustained activation of the cyclic adenosine 3′, 5′-monophosphate (cAMP) system in specific brain regions [146].

#### 2.6.1. Serotonin Sensing

Neurotransmitter metabolites such as 5-hydroxyindoleacetic acid (5-HIAA) and cortisol exhibit significant changes in patients with depression. 5-HIAA is a primary metabolite of serotonin (5-HT) and is mainly excreted through urine. Given that serotonin plays a crucial role in mood regulation, changes in 5-HIAA levels in depression suggest dysfunctions in the serotonin system [147].

**Electrochemical Sensing**

He et al. utilized the photocatalytic activity of TiO_2_ and the conductivity of Ag nanoparticles to fabricate a multi-level TiO_2_-Ag nanocomposite material [148]. They polymerized a P(VPA-SBMA-GMA) hydrogel antifouling layer on the surface of the nanocomposite and further functionalized it with 5-HT aptamers for specific recognition. The resulting sensor demonstrated a broad detection range for 5-HT (0.5 pM to 100 nM) and a low detection limit (5 fM). Moslah et al. developed a portable electrochemical sensor for serotonin (5-HT) based on environmentally friendly silver nanoparticles and reduced graphene oxide (AgNPs-rGO)-modified screen-printed carbon electrodes (SPCEs) [149]. Liao et al. created a wearable serotonin sensor using graphite ink and multi-walled carbon nanotubes, achieving a minimum detection limit of 45 nM [150].

Li et al. screened aptamers for 5-HT sensing and developed an electrochemical sensor with an LOD of 0.3 μM [151]. Further improvements by Li et al. reduced the LOD to 2 nM [152]. They established a sensitive and selective voltammetric biosensor on a screen-printed carbon electrode with gold nanoparticles deposited on it, utilizing a layer-by-layer assembly of positively charged poly(diallyldimethylammonium) (PDDA-oSWCNTs), negatively charged 5-HT-specific aptamers, and tyrosinase. Zhan et al. combined molecular imprinting with bimetallic-functionalized probe sensing to detect 5-HT [153]. Compared to single-metal interfaces or molecularly imprinted layers alone, the synergistic microbial sensor exhibited superior performance for 5-HT detection, with 5-HT being adsorbed and catalytically oxidized by the imprinted cavities.

**Optical Sensing**

In the realm of optical sensing, various advancements have been made. Avci et al. developed a colorimetric sensor for serotonin (5-HT) [154]. This sensor leverages the inherent binding affinity of serotonin to sialic acid molecules anchored on gold nanoparticles (SA-AuNPs). Upon binding of 5-HT, SA-AuNPs aggregate, causing a red shift in the absorbance spectrum of SA-AuNPs, which leads to a significant color change. This change can be measured spectroscopically, with other biomolecules showing no color change. The sensor demonstrated high selectivity, sensitivity, and rapid response, with a detection range of 0.05 to 1.0 μM, a detection limit of 0.02 μM, and a response time of 5 min.

Ryu et al. utilized target-specific aptamers at the aqueous/liquid crystal (LC) interface, decorated with cationic surfactants, for serotonin detection [155]. In increased 5-HT levels, the specific binding of 5-HT to the aptamers reduced the interaction between the aptamers and cetyltrimethylammonium bromide (CTAB), maintaining the alignment of the LC molecules. The orientation transition of LCs was observed under a polarized optical microscope. This sensor had a linear detection range from 1 to 1000 nM, with a detection limit of 1.68 nM.

Zhang et al. developed a fluorescence-based serotonin sensor that integrates with a smartphone [156]. They employed Thioflavin T (ThT) as the dye molecule in the system, utilizing the binding affinity differences between the aptamer, 5-HT, and the dye. When 5-HT is introduced, it restricts the rotational motion of ThT molecules, resulting in strong fluorescence. In the presence of serotonin, the aptamer further folds, releasing the bound dye molecules and diminishing the fluorescence of ThT. Real-time detection of serotonin in complex biological fluids using UV light as an illumination source and capturing sample droplets with the iPhone 13. This sensor showed a detection range of 0.4 to 2 μM and a detection limit of 19 nM.

#### 2.6.2. Dopamine Sensing

Dopamine (DA) is another critical biomarker closely associated with depression. As a precursor to norepinephrine and epinephrine, dopamine is itself a neurotransmitter and is thought to be closely linked with motivation and reward in the brain [157,158,159,160]. Reduced dopaminergic neurotransmission has been implicated in MDD, and manipulating DA neurotransmission is an important therapeutic approach for depression [161,162,163]. Dopamine sensing studies are more abundant, especially regarding the modification methods of the electrodes. The relevant studies are summarized in Table 1.

**Electrochemical sensing**

Electrochemical methods are commonly used for dopamine sensing, which often involve modifying traditional electrodes (such as glassy carbon electrodes) and designing special sensor structures to amplify the signal. Various studies have employed diverse electrode modification strategies to achieve desirable detection metrics. For instance, Elugoke et al. reported the use of poly(2,4,6-trihydroxybenzaldehyde) (PTGCE) electrodeposited on bare glassy carbon electrodes for dopamine detection [164]. Abraham et al. used atomic-layer deposition (ALD) to prepare hafnium oxide (HfO_2_) nanofilms on silicon (Si) for fabricating non-enzymatic electrochemical dopamine sensors with selective and sensitive detection capabilities [165]. Due to the hydrophobic interactions between the HfO_2_ nanofilm and dopamine, and the electrostatic attraction between the negatively charged hydroxyl groups on the membrane surface and the positively charged amine groups of dopamine, the HfO_2_ nanofilm selectively serves as a local dopamine binding site.

Shahparast and Asadpour-Zeynali developed an environmentally friendly electrochemical sensor using CuAl-layered double hydroxide (LDH)/GCE, synthesized through a simple one-step co-precipitation method [166]. LDH carries a negative charge and has a high electrostatic attraction for dopamine while repelling negatively charged ascorbic acid and uric acid, thus providing some specificity for dopamine detection. Doan et al. developed a high-performance non-enzymatic electrochemical sensor based on a 3D porous copper foam-supported sea urchin-like CoP_3_/Cu_3_P heterostructure nanorod, where the sea urchin-like microsphere structure provided a large electrochemically active surface area [167].

Zhang et al. synthesized a nanocomposite (BC@Cu-BTC) composed of copper-based organic frameworks (Cu-BTC) and bamboo charcoal (BC) [168]. The large specific surface area of Cu-BTC (1245.25 m^2^/g) and its synergistic effect with BC enhance the current signal for detecting dopamine in aqueous solutions. Zhang et al. also prepared ZnO-CeO_2_ hollow nanospheres using a hard template method, where CeO_2_ acts as a supporting framework to prevent ZnO agglomeration [169]. The high specific surface area and synergistic effects of ZnO and CeO_2_ confer the synthesized ZnO-CeO_2_ hollow nanospheres with a large electrochemically active area and high electron transfer rate. Keerthanaa et al. proposed a wearable electrochemical sensor based on microneedles for continuous monitoring of dopamine in interstitial fluid (ISF) [170]. They used a chitosan-protected hybrid nano-Fe_3_O_4_-GO composite as the chemical recognition element and applied a Nafion antifouling coating for protection.

**Figure 8:**
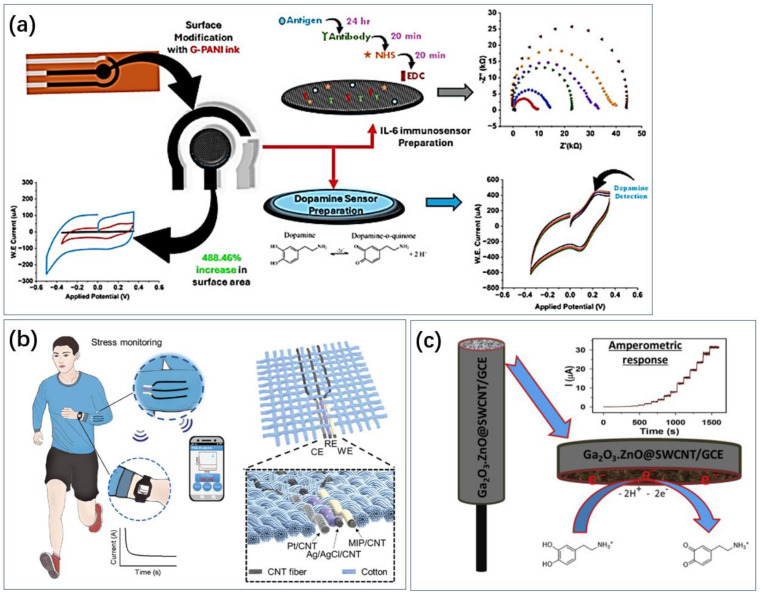
Applications of carbon-based metamaterials. (**a**) Laser-induced graphene has a large specific surface area and the prepared electrodes also have good flexibility and can be used for dopamine sensing [96]. Reproduced with permission, Copyright 2024 American Chemical Society (**b**) and single-walled carbon nanotubes have a large specific surface area and stability and are also often used in the development of electrodes for cortisol sensing [178]. Reproduced with permission, Copyright 2024 WILEY—V C H VERLAG GMBH & CO. KGAA (**c**) Carbon nanotubes assembled into fibers have good flexibility and can be combined with everyday clothing for cortisol sensing [173]. Reproduced under the terms of the Creative Commons Attribution License, Copyright 2023 by the authors, published by Elsevier.

Carbon materials, such as carbon nanotubes and graphene, are widely used for electrode modification due to their good specific surface area and abundant binding sites (Figure 8). Ghosh et al. also realized electrochemical sensing of DA using electrodes made from graphene conductive ink (Figure 8a) [94]. Singh et al. used carbon nanotube-encapsulated nickel selenide composite nanostructures as non-enzymatic electrochemical sensors for detecting dopamine [171]. Aldughaylibi et al. developed modified electrodes based on molybdenum oxide (MoO_3_) grown on graphite sheets (GSs) [172]. Ahmed et al. utilized Ga_2_O_3_-doped ZnO-modified SWCNT (Ga_2_O_3_⋅ZnO@SWCNT) nanocomposites to modify glassy carbon electrodes (Figure 8c) [173]. Compared to pure ZnO, the nonstoichiometry and oxygen vacancies in the doped oxide impart superior conductivity to the Ga_2_O_3_⋅ZnO@SWCNT nanocomposites. Jaryal et al. synthesized nitrogen-functionalized MWCNTs (TM-CNT600) by thermally annealing carboxyl-functionalized MWCNTs with thiourea at 600 °C under a nitrogen atmosphere [174]. Xi et al. constructed a novel electrochemical sensing electrode modified with hydrophilic hyaluronic acid (HA) and curcumin/multi-walled carbon nanotubes (CM/MWCNTs) [175]. Due to the inherent hydrophilicity of HA, the constructed electrochemical sensor for dopamine detection exhibited significant protein adsorption resistance. The synergistic effect of the CM/MWCNT composite provided excellent electrochemical behavior (quinoid/benzoid redox pairs). Compared to bare GCE, MWCNTs/GCE, and CM/MWCNTs/GCE, the electrocatalytic current for dopamine on HA/CM/MWCNTs/GCE increased by 5.0-, 3.5-, and 2.4-times, respectively. Yashil and Koçoglu developed an amperometric dopamine sensor based on glassy carbon electrodes (GCEs) modified with carbon nanofibers (CNFs), Fe_3_O_4_ nanoparticles (Fe_3_O_4_NPs), and silver nanoparticles (AgNPs) [176]. Kabas prepared an ultrasensitive electrochemical sensor for detecting dopamine (DA) based on a composite of palladium nanoparticles/4-aminophenol-functionalized nitrogen-doped graphene quantum dots (PdNPs/4AP N-GQDs) [177].

Other 2D materials and MIP methods are also widely applied in dopamine sensing. Xiao et al. constructed an electrochemical sensor based on a ternary composite of 1D Pt nanowires, 2D MXene nanosheets, and 3D porous carbon [179]. Pt nanowires, with their abundant grain boundaries and highly undercoordinated atoms, exhibit excellent catalytic activity; MXene nanosheets not only facilitate the growth of Pt nanowires but also enhance their conductivity and hydrophilicity; porous carbon contributes to the significant adsorption of dopamine on the electrode surface. Chakraborty et al. developed a flexible sensor using 2D cobalt telluride (2D CoTe_2_), based on dopamine adsorption on 2D CoTe_2_ [180]. Additionally, flexible paper-based sensors made from 2D CoTe_2_ have been successfully used for the real-time detection of dopamine in artificial sweat, with an LOD of 0.22 pM. Mabrouk et al. used PY and CTS as functional materials and dopamine as a template to electropolymerize a composite material on the surface of GCE modified with TiO_2_-prepared NPs, establishing an MIP matrix for dopamine detection [181].

**Optical Sensing**

Apart from the aforementioned electrochemical methods, optical sensing techniques can also be applied for dopamine detection, commonly utilizing fluorescence and SPR technologies. Jabbari et al. developed a dopamine biosensor based on SPR, using a carboxymethyl dextran SPR chip to immobilize laccase as a bioaffinity recognition element [182]. The kinetic affinity (KD) of 48,545 nM, calculated through molecular docking studies, indicated a strong association between dopamine and the laccase active site. Kayalik and Saçmaci established a novel platform for dopamine detection using surface-enhanced Raman scattering (SERS) technology, employing a CeO_2_@TiO_2_ nanocomposite glass substrate with polyethylene glycol, AuNPs, and AgNP nanomaterials [183]. Sharma et al. proposed an innovative fiber optic biosensor based on the LSPR effect for high sensitivity and selective dopamine detection [184]. The biosensor probe employed a single-mode fiber-multimode fiber-single-mode fiber (SMS) structure, chemically modified to enhance the LSPR effect. Gold nanoparticles were used to amplify the plasmonic response, thereby improving the sensing performance. Similarly, Vikas et al. reported a highly sensitive and selective SPR sensor for dopamine detection, modified with small copper oxide nanoparticles (CuO NPs) [185]. A 50 nm thick gold film was deposited on the uncoated portion of a multimode fiber via magnetron sputtering, and the fiber sensor probe was further modified with synthesized CuO NPs (~7 nm).

Sliesarenko et al. developed a simple dopamine detection method using an LED and o-phthalaldehyde (OPA) as an indicator [186]. The fluorescence signal of the dopamine-OPA reaction was tested with three Pluronics as additives, with Pluronic F127 resulting in a 16-fold increase in fluorescence. Tian et al. prepared copper nanoclusters (Cu NCs) using polyvinylpyrrolidone (PVP) as a protective ligand and L-ascorbic acid (L-AA) as a reducing agent through a simple hydrothermal method [187]. The prepared PVP Cu NCs exhibited strong blue emission at 427 nm when excited at 365 nm. Under alkaline conditions, dopamine selectively quenched the fluorescence of PVP Cu NCs. Mechanistic studies showed that dopamine self-polymerized to form polydopamine, which inhibited the emission of PVP Cu NCs at 427 nm through an inner filter effect (IFE). Based on this phenomenon, a simple and highly selective method for dopamine determination was established.

**Figure 9:**
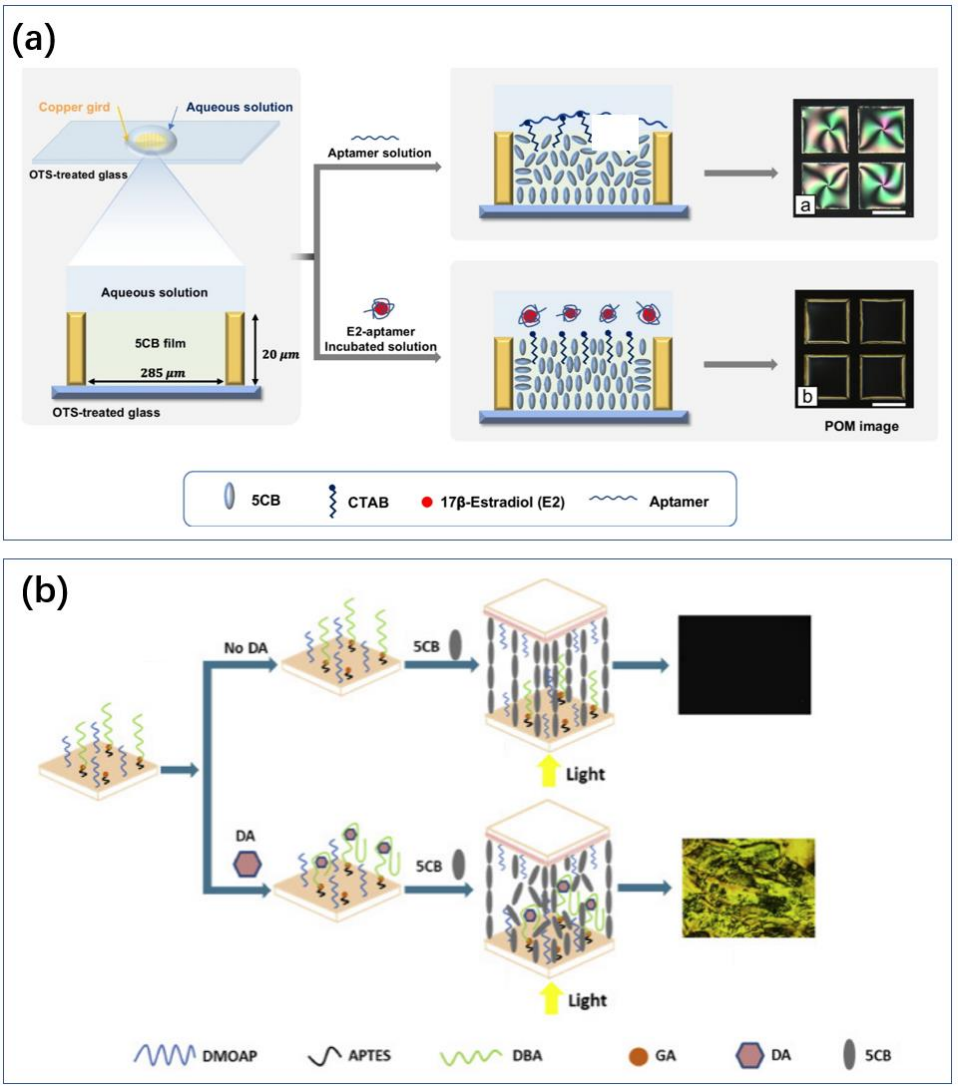
Different ideas for liquid crystal sensors. (**a**) The aptamer is pre-positioned at the LC-water interface and the LC molecules resume alignment after the marker attracts the aptamer [191]. Reproduced with permission, Copyright 2023 Springer-Verlag GmbH, DE part of Springer Nature. (**b**) LC molecules are originally well aligned and the entry of marker and aptamer disrupts the molecular alignment [188]. Reproduced under the terms of the Creative Commons Attribution License, Copyright 2020 by the authors, published by Elsevier Inc.

Finally, the liquid crystal method has also been used for dopamine sensing. Nguyen immobilized dopamine-binding aptamers (DBAs) on the surface of slides with glutaraldehyde and achieved sensing by disrupting the orientation of LC molecules using DA binding to DBAs (Figure 9b) [188]. It can be successfully applied to detect DA in human urine without labeling. Nandi et al. used gold nanoparticles to amplify the signal of a dopamine liquid crystal biosensor [189]. The functionalized gold nanoparticles were attracted to the surface of the liquid crystals, leading to a greater change in the arrangement of the liquid crystal molecules. In contrast to the approach of Huang et al., the pre-positioned aptamer disrupts the arrangement of the liquid crystal molecules (similar principle to Figure 9a) [190]. The binding of dopamine to the aptamer enhances the assembly of the molecules at the LC–water interface.

**Table 1:** Summary of dopamine sensing technologies.

Type	Method	Probes/Substrates	LOD	Sensing Range	Ref.
Electrochemical	Modified electrodes	Ga_2_O_3_⋅ZnO@SWCNT/GCE	0.052 μM	1.0–2056 μM	[173]
		MoO_3_/GS	2.71 nM	1–10 nM	[172]
		PTGCE/GCE	0.64 μM	0.70–19.48 μM	[164]
		2D CoTe_2_/GCE	0.21 pM	/	[180]
		TM-CNT600/GCE	1.42 μM	10.7–24.2 μM	[174]
		ITO/glass substrate + CuO/CuO_2_	0.388 μM	0–20μM	[192]
		CuAl-LDH/GCE	0.33 μM	4.194–1151.54 μM	[166]
		HA/CM/MWCNTs/GCE	0.009 μM	50–200 μM	[175]
		AgNP/CNF−Fe_3_O_4_NP/GCE	0.18 μM	0.2–550μM	[176]
	MIP	TiO_2_ NPs/GCE	0.281μM	1–10μM	[181]
	Microneedles	Fe_3_O_4_-GO/CP	90 nM	/	[170]
	Composite materials	PdNPs/4AP N-GQDS	21 pM	250 pM–10 nM	[177]
		NiSe_2_@CNT	/	5 nM–640 μM	[171]
		Pt NWs/MXene/porous carbon	28 nM	0.1–200.0 μM	[179]
		BC@Cu-BTC	1.572 μM	1–100 μM	[168]
	Nanofilms	HfO_2_-200/Si	0.4 pM	0–1000 pM	[165]
	Nanorods	CoP_3_/Cu_3_P NRs/CF	0.51 mM	0.2–2000 mM	[167]
	Hollow Nanospheres	ZnO-CeO_2_	0.39 μM	5–800 μM	[169]
	Aptamer electrodes	Aptamer/CFE	88 nM	0.2–20 μM	[193]
Optical	LSPR	Laccase	0.1 ng/mL	0.01–189 μg/mL	[182]
		SMS optical fiber	/	400 nM–50 μM	[184]
		Multimode fiber/gold film/CuO NPs	1.43 nm	1.11 nM–50 nM	[185]
	Surface-enhanced Raman Scattering (SERS)	CeO_2_@TiO_2_ nanocomposite terminated glass substrate/polyethylene glycol/AuNPs/AgNPs	0.01 pM	1 pM–1 M	[183]
	Fluorescent	OPA	0.015 µM	0.5–3 µM	[186]
		Cu NCs/PVP/L-AA	1.32 μM	5–200 μM	[187]
	liquid crystal	DBA/Glutaraldehyde/DMOAP	10 pM	1 pM-10 μM	[188]
		5CB/3NPBA/DSP-GNP	0.3 μM	0.1–1.0 μM	[189]
		5CB/CTAB	2.51 pM	10 pM-1 μM	[190]

#### 2.6.3. Acetylcholine Sensing

Acetylcholine (ACh) is another important neurotransmitter, abundant in the human brain but decreasing with age. Its pathways interact with dopamine, influencing the occurrence of depression [194]. Increased ACh signaling can lead to symptoms associated with anxiety and depression, and, while acetylcholine is also a target for Alzheimer’s disease treatment, its monitoring during therapy is crucial [195,196]. Targeting nicotinic acetylcholine receptors in the brain is a strategy for treating depression comorbid with addiction [197]. Because acetylcholine and its corresponding enzyme acetylcholinesterase are also important regulators in plants, extensive research focuses on detecting their levels in environmental and water samples. This review, however, concentrates on acetylcholine sensing in human samples and portable or wearable biosensing technologies.

Firstly, regarding the design of electrochemical sensors, acetylcholinesterase can catalyze the breakdown of acetylcholine, leading to a series of electrochemical sensors using acetylcholinesterase as a probe. Chen et al. covalently immobilized acetylcholinesterase on a gold microelectrode surface via a disulfide-based cross-linker, achieving a sensing range of 5.5–550 μM [198]. Jing et al. developed an electrochemical biosensor for ACh detection using a glassy carbon electrode (GE) modified with dual-enzyme-functionalized nanofiber composites [199]. The electrospinning process significantly increased the surface area. Sensors using acetylcholinesterase as a substrate can also measure concentrations of anti-acetylcholinesterase drugs, useful for monitoring blood levels of Alzheimer’s disease medications, which could prevent or exacerbate depression [200]. However, this application is not directly related to depression and is not further elaborated in this review.

Zhang et al. designed an optical sensor for acetylcholine detection using acetylcholinesterase [201]. They immobilized acetylcholinesterase on an optical fiber for label-free ACh detection, achieving a detection limit of 30 nM with temperature compensation. Zhang et al. improved the sensitivity of refractive index sensing by immobilizing gold nanorods on the surface of light rays and further utilized 1,6-hexose dithiol as a cross-linking agent instead of electrostatic adsorption in the following year to reduce the final sensor LOD to 0.45 μ g/mL [202,203].

Due to the potential inactivation of enzymes in vitro, some studies focus on electrochemical sensors using composite catalysts and artificial substrates. Pitiphattharabun et al. developed electrodes using graphene materials [204]. The composite of graphene oxide (GO) and reduced graphene oxide with zinc oxide (rGO/ZnO) enhanced electron transfer efficiency, enabling ACh sensing. Poolakkandy et al. proposed a non-enzymatic electrochemical sensor for ACh detection based on a copper–cobaltite/MWCNT composite, successfully fabricating a flexible sensor [205]. Wen et al. successfully loaded Pt nanoparticles (Pt NPs) onto a Zr-based metal–organic framework (MOF-808) [206]. This composite catalyst effectively mimicked the functions of acetylcholinesterase (AChE) and peroxidase (POD). Utilizing this capability, they constructed a sensitive biosensor for ACh detection, amplifying the signal through the oxidation of a chromogenic substrate by changing the solution’s pH, driven by ACh hydrolysis.

Since acetylcholine is easily decomposed by acetylcholinesterase and is related to real-time neural signaling in the brain, current methods like positron emission tomography (PET) and magnetic resonance spectroscopy (MRS) are used for testing but are not ideal for long-term self-monitoring by patients [207]. Therefore, much research focuses on implantable in vivo biosensors. Amirghasemi et al. designed a flexible electrochemical sensor for implantable biosensors [208]. They used a cotton yarn with a diameter of 250 μm coated with flexible conductive ink and a calcium alkyne ion group containing the ACh sensing membrane. The overall diameter of the sensor is 400 μm, with good flexibility and an LOD of 20 μM due to the background signal from cerebrospinal fluid ions. Sudalaimani et al. used common pipette tips to create a disposable liquid-phase electrochemical sensor [209]. Electrochemistry at the interface of two immiscible electrolyte solutions (ITIES) has garnered attention for molecular sensing without recognition elements, overcoming the contamination, nonspecific adsorption, and stability issues often encountered with modified electrodes. Using a pre-pulled glass micro-pipette, they developed a method for sensing acetylcholine at the liquid–liquid interface. In both methods, during the backward transfer of acetylcholine, the liquid–liquid organogel and liquid–liquid interface configurations showed a linear increase in current.

#### 2.6.4. Partial Summary in Neurotransmitter Sensing

Neurotransmitters are a very-small-molecular-weight class of the markers summarized here. They are an ideal class of markers due to their strong correlation with nervous system activity and their association with a variety of depression etiologies and pharmacologic treatment mechanisms. However, due to their small molecular weight, the specificity of sensing using inorganic probes needs to be further explored, and there is a wealth of reports on this (Table 1).

There are fewer options for organic probes. Sensing using enzymes has good specificity and sensitivity, but enzyme inactivation can lead to challenges in device shelf life and integration with the rest of the sensing. Finally, in contrast to hormones, which can be widely diffused throughout the body in blood and even urine and sweat, neurotransmitters are metabolized between synapses. The collection of cerebrospinal fluid is more specialized and painful, while the association between neurotransmitter concentrations and neural activity is weakened in blood and non-invasive body fluid samples. Some studies have also turned to measuring neurotransmitter metabolites in easily collected samples.

### 2.7. Remaining Markers

Some of the substances that can be used in the diagnosis of depression, such as IL-8, are also mentioned in the related background introduction.

**Figure 10:**
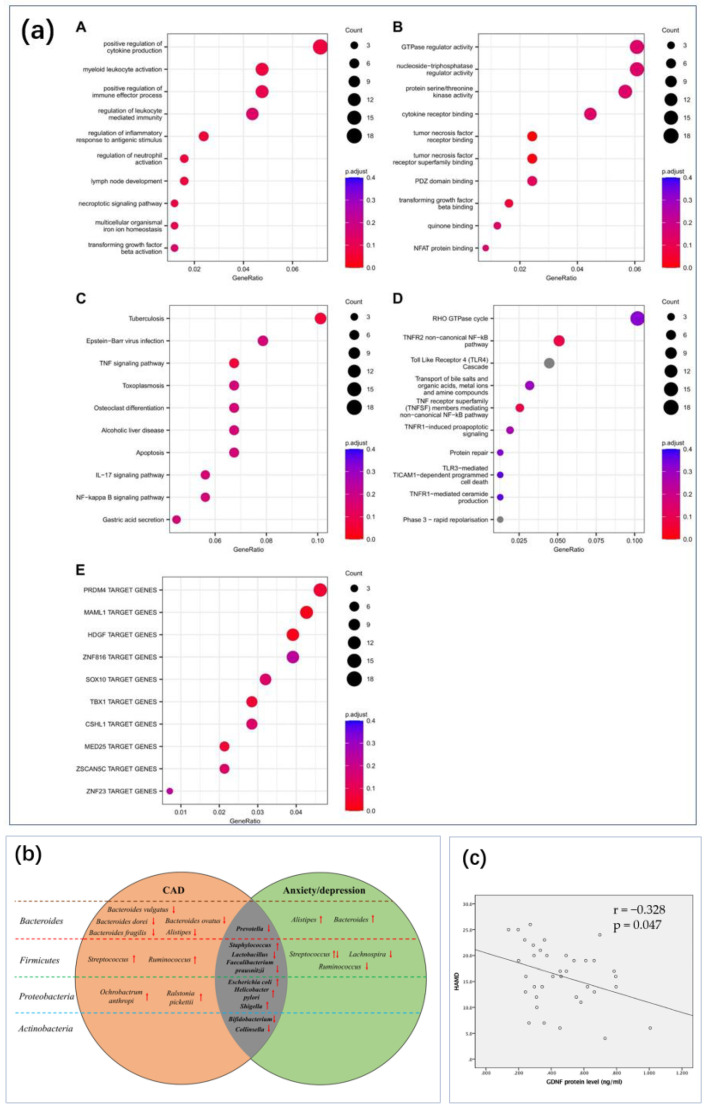
Depression markers that require further research on sensing technologies. (**a**) Gene set enrichment analyses on the genes annotated to significant differentially methylated regions in the longitudinal analysis performed between T0 and T12, in patients who underwent EMDR. Top 10 enriched gene sets for (A) GO BP and (B) GO MF. Top 10 enriched pathways for (C) KEGG and (D) Reactome. (E) Top 10 enriched Transcription Factor Target gene set defined by the MSigDB (collection C3: regulatory target gene sets, GTRD subset) [213] Reproduced under the terms of the Creative Commons Attribution License, Copyright 2024 by the authors, published by Informa UK Limited (**b**) The altered tendency for GM of CAD and anxiety and depression from the four aspects of Firmicutes, Bacteroidetes, Proteobacteria, and Actinobacteria [214]. Upward and downward arrows indicate an increase or decrease in the number of bacteria in the situation of anxiety and depression, respectively. Reproduced under the terms of the Creative Commons Attribution License, Copyright 2021 by the authors, published by Aging and Disease (**c**) The correlation analysis showed that GDNF protein level negatively correlated with the value of HAMD-17 in PSD patients (correlation coefficient  =  −0.328, *p*  =  0.047). The slash is the result of the fit. Abbreviations: GDNF, glial cell line-derived neurotrophic factor; HAMD, Hamilton depression rating scale; PSD, post-stroke depression [210]. Reproduced under the terms of the Creative Commons Attribution License, Copyright 2017 by the authors, published by Springer Nature.

In addition to these, glial cell-derived neurotrophic factor (GDNF) has been found in a variety of cultures of neuronal and neural-related cells. Many studies confirm the association between its concentration and depression (Figure 10c) [210]. For GDNF and its pathways, several studies have reported the therapeutic ideas that can be used to alleviate depression. However, regarding the markers, there are fewer relevant sensing studies. Although studies are focusing on biosensing technologies, they are far less abundant than the markers summarized in the main text, and there is a lack of convenient technological pathways to explore [211]. The metabolism of glucose, a nutrient for cells in the body, can also reflect depression levels. Fan et al. reported the involvement of O-GlcNAc transferase (OGT) in the metabolism of glucose and that its transcript RNA appeared to be elevated in patients with depression and demonstrated the potential for therapeutic use [212]. The number of studies on sensing based on various cellular nutrients that help to respond to the metabolic level of neuronal cells and are used to aid in the diagnosis of depression is still small.

Additionally, out of many hormones, therefore, they can be used as markers, and the receptors used to receive them and the genes encoding them can be used as diagnostic markers. In particular, gene polymorphisms, methylation, etc., will indirectly affect the expression level of the relevant transcription proteins, which may be the cause of depression (Figure 10a) [213]. Their detection also enables the diagnosis of depression and may further explain the genetics behind the biochemical diagnosis. The 5-Hydroxytryptamine transporter gene polymorphism (5-HTTLPR) is also a marker of great interest, with the 5-HTTLPR genotype being associated with MDD but not with suicide or 5-HTT binding and 5-HTTLPR moderating the relationship between stress and depression [215,216]. Similarly, there is a genetic polymorphism marker associated with BDNF (BDNF Val66Met), which involves a variant in amino acid 66 of the BDNF gene that affects BDNF secretion and function, and the BDNF Val66Met polymorphism has a significant effect on MDD in males [217].

DNA methylation has also been an important area of research, with DNA methylation being the process by which a DNA molecule adds methyl groups to cytosine residues, a modification that regulates the transcriptional activity of genes. It has been found that the promoter regions of certain genes may be abnormally methylated in patients with depression, which, in turn, affects the expression of related proteins and ultimately leads to depression [218,219]. Similar sensing of depression based on genetic material or non-coding RNA (ncRNAs) could help to reveal the pathogenesis at a deeper level. Portable or wearable biosensing technologies for these genes are less well studied overall. Related research advances focus, in part, on rapid detection to address POC needs. The goal is to enable rapid and accurate diagnosis to provide targeted treatment in hospital scenarios, and sensing of such markers was systematically summarized in an article by Dong et al. [220].

Finally, gut microbes have also shown correlations with depression and anxiety (Figure 10b) [214].

### 2.8. Summary in Biochemical Sensing

Depression is a systemic, complex disorder with large individual differences. It places a significant burden on both the patient’s health and the healthcare system. To achieve personalized treatment, richer sensing technologies have important implications. Much of the current research is pushing towards portability and wearability (summarized in Table 2). Electrochemical sensors are highly versatile; electrodes are often easier to miniaturize than optical systems and can be conveniently fabricated into arrays and converted into computer-readable signals. Many marketed products have been reported [221]. However, there is still a lack of consumer-grade products reported for depression. The optical law has demonstrated, in many studies, its potential for rapid screening without the need for specialized instrumentation, through simple colorimetric methods such as colorimetry.

**Table 2:** Biochemical sensing research with advances in portability and wearability.

Progress	Marker	Type	Probes/Substrates	LOD	Sensing Range	Ref.
Wearable sticker	Cortisol	Electrochemical	Antibodies + extended-gate AlGaN/GaN high electron mobility transistor (HEMT) + sapphire substrate	100 fM	1 nM–100 μM	[32]
Smartphone	Cortisol	Fluorescent	Rhodamine	~nm	1 mM–1 pM	
Microneedle patch	Cortisol	Fluorescent	Europium metal−organic frameworks (Eu-MOF)	1 nM	0.1 μM–1 mM	
Mobile phone/smart watch	Cortisol	Electrochemical	MIP/CNT + fabric sensing system (FSS)	1 pM	1 pM–10 μM	[178]
Naked eye	Thyroxine	LSPR	Gold triangular nanoplates (AuTNPs)	200 nM	0.02–5 μM	[49]
Smartphone + miniaturized potentiostat (M-P)	Testosterone	Electrochemical	PoPD-MIPs/SPCE	1 ng/dL	1–25 ng/dL	[64]
Portable test swabs + naked eye	Melatonin	Colorimetric/luminescence	Fe/Zn/Ir TAzyme	Ccolorimetric: 8.9 nM luminescence: 8.8 nM	0.01–500 μM	[222]
Smartphone	Melatonin	Fluorescent	Blue-emissive carbon dots (BCDs)/C3N4 nanosheets loaded with platinum/ruthenium nanoparticles (PtRu/CN)/OPD/H2O_2_	23.56 nM	0.06–600 μM	[48]
Band-aids	Melatonin	Electrochemical	Zn-MOF-Nb2CTx Mxene/carbon yarn (CY)	215 nM	1–100 μM	[74]
Smartphone	Melatonin	Fluorescent	3,6-Diaminocarbazole (DAC)	1.46 µM	0–78 μM	[75]
Smartphone + NFC microchip	CRP	Electrochemical	Anti-CRP Nanobodies/Screenprinted graphene electrodes (SPGE)	1.18 ng/mL	0.01–100 μg/mL	
Wearable devices	IL-6	Electrochemical	LIG/G-PANI electrodes	2.6234 pg/mL	0.002–20 pg/mL	[96]
	Dopamine		LIG/G-PEDOT:PSS electrodes	0.567 μM	0.5–5 μM	
			LIG/G-PANI electrodes	0.4084 μM	0.5–5 μM	
Portable devices	Dopamine	Electrochemical	Graphene conductive polymer paper-based sensor (GCPPS)	3.4 µM	12.5–400 µM	[223]
	TNF-α			5.97 pg/mL	0.005–50 ng/mL	
	IL-6			9.55 pg/mL	2 pg/mL–2 µg/mL	
Miniaturized portable devices	IL-6	Electrochemical	Boron nitride nanosheet/gold nanoparticle (BNNS/AuNP)/SPCE + anti-IL-6	5 pg/mL	0.01–200 ng/mL	[97]
Integrated portable devices	IL-6	Photoelectrochemical	AuNPs@ dsDNA/CS/CdS QDs/ZnO NSs@OF (ADCCZ@OF)	0.19 pg/mL	1–100 pg/mL	[103]
Wearable biosensor	Serotonin	Electrochemical	Graphite sheet/graphite ink (GI)/multi-walled carbon nanotube (MWCNT)	45 nM	100–900 nM	[150]
Portable biosensor	Serotonin	Electrochemical	AgNPs-rGO/SPCE	5.25 μM	10–200 μM	[149]
	Dopamine			4.36 μM	10–200 μM	
	Serotonin & dopamine simultaneously			serotonin: 7 μM dopamine: 7.41 μM	10–100 μM	
Smartphone + portable biosensor	Serotonin	Fluorescent	5-HT aptamer/ThT	19 nM	0.4–2 μM	[156]
Portable biosensor	Dopamine	Electrochemical	Paper-based 2D CoTe_2_/GCE	0.22 pM	/	[180]
Wearable microneedle-based electrochemical sensor	Dopamine	Electrochemical	Fe_3_O_4_-GO/chi/carbon paste-filled hollow microneedles	90 nM	3–32 μM	[170]

Depression has a wide variety of causes and manifestations. It is difficult to draw simple conclusions about what combination of markers is most conducive to diagnosis. There are also large differences in sensing principles between depression markers, from microbes and DNA to large organic molecules and small-molecule markers. However, there is no single marker that can achieve good monitoring of depression, and more systems and algorithms that integrate multiple markers are needed to provide a richer diagnosis for each patient. There are fewer studies on combining different classes of chemical sensors (Table 3). This may be because quantitative comparisons of multi-sensor combinations for depression diagnosis are difficult compared to single sensors that can compare LoD. The lack of relevant data further hinders the advancement of research on sensor combinations. Perhaps, in the future, it will be possible to focus on some of the common types of depression and give several biochemical sensing options that will help in the monitoring of eligible patients.

**Table 3:** Biochemical sensing combinations of different markers.

Detectable Biomarkers	Detection Method	LOD	Sensing Range	Ref.
Tyr; D-Tyr	GQDs and β-CDs modified GCE	6.07 nM and 103 nM	\	[224]
Serotonin; Dopamine	GO and 5,15-pentafluorophenyl-10,20-p-aminophenylporphyrin	3.5 × 10^−2^ μM and 4.9 × 10^−3^ μM	\	[225]
Serotonin; Dopamine	Electrografting-assisted site-selective functionalization of aptamers on graphene field-effect transistors (G-FETs)	10 pM (Dopamine)	10 pM–100 μM	[226]
Serotonin; Dopamine and AA	Graphene and poly 4-amino-3-hydroxy-1-naphthalenesulphonic acid deposited on the surface of carbon-based SPE	2.4 nM, 2.8 nM and 160 nM	0.01–150 μM, 0.01–120 μM and 0.5–100 μM	[227]
Dopamine, epinephrine and serotonin	A film electrode entirely composed of oppositely charged carbon nanoparticles	0.4 mM, 1.0 mM and 0.8 mM	0.4–350 mM, 1–49 mM and 0.8–100 mM	[228]
All nine essential AAs as well as vitamins, metabolites and lipids commonly found in human sweat	Two carbachol-loaded iontophoresis electrodes, a multi-inlet microfluidic module, a multiplexed MIP nutrient sensor array, a temperature sensor and an electrolyte sensor	702 nA mm^−2^ per decade of concentration	\	[229]
glucose, lactate, uric acid, sodium ions, potassium ions and ammonium	Carbachol hydrogel-loaded sweat-stimulation electrodes, three enzymatic biosensors, three ion-selective sensors (ISEs)	98.7% classification rate	\	[230]

## 3. Wearable Physiological Signal Sensing

Treatment for depression is a long-term process. Regular monitoring is essential to ensure patient health and safety [231]. For patients with a history of recurrent episodes, maintenance therapy may last for one year or longer and, sometimes, even for a lifetime to prevent relapse. During this period, patients often return to their normal lives outside the hospital. Statistical data indicate that the suicide risk among individuals with depression is significantly higher than that of the general population, with the suicide rate of depression patients approximately 20-times higher. About 15% of patients with MDD attempt suicide, and 5% to 10% ultimately die by suicide.

Another important aspect of monitoring during the treatment process is to improve therapeutic strategies more effectively. Due to the complex etiology of depression and the intricate nature of drug actions and side effects, especially symptoms like guilt and suicidal ideation, which are difficult to reproduce in animal models, pharmacological animal studies often fall short [147,232]. The use of antidepressant medications requires adjustment based on patient responses and side effects. Treatment usually begins with a low dose, gradually increasing according to the patient’s tolerance and response. During treatment, physicians regularly assess symptoms and medication side effects, adjusting doses or changing medications as needed. Sometimes, a single medication may be insufficient, necessitating the combination of multiple drugs or adjunctive treatments such as psychotherapy. If a patient’s condition stabilizes and medication discontinuation is considered, doctors generally reduce the dose gradually to prevent withdrawal symptoms and relapse.

With advancements in wearable electronics, it has become possible to continuously monitor physiological signals related to depression for feedback on treatment adjustments and early warning of extreme emotions. Long-term monitoring of these physiological and behavioral parameters allows for a more comprehensive assessment of treatment efficacy, provides early warning mechanisms, and helps prevent the deterioration of depressive symptoms or the occurrence of extreme events.

### 3.1. Heartbeat Monitoring

Heart rate variations can reflect psychological and emotional states. Depression patients often experience tachycardia or reduced heart rate variability (HRV), which is attributed to dysregulation of the autonomic nervous system. Tachycardia is typically associated with excessive activation of the sympathetic nervous system, while reduced HRV indicates decreased activity of the parasympathetic nervous system. These changes are closely related to psychological stress and anxiety. Some antidepressant medications can also cause changes in electrocardiograms (ECGs); for example, in an 11-year-old case, the clinical response to amitriptyline was complicated by ECG changes, leading to abrupt discontinuation of the medication and the development of withdrawal syndrome [233].

One common non-invasive method for measuring and recording cardiac electrical fluctuations is ECG. Traditional 12-lead ECG systems use ten Ag-AgCl electrodes placed at specific locations on the body to measure cardiac electrical activity in twelve specific directions. Wearable devices typically use fewer electrodes, which can be broadly categorized into wet electrodes (gel electrodes) and dry electrodes. Key considerations in electrode design include ultra-thinness, high conformability, stretchability, low modulus, durability, breathability, and elimination of motion artifacts. Lu et al. proposed a wearable depression monitoring system utilizing a specific system-on-a-chip solution that achieved filtering and feature extraction of HRV from ECG. Using a smartphone application, this system trained and classified users’ depression scales with an accuracy of 71% [234]. Huang et al. used only three electrode attachment points on the chest, combined with ZigBee for wireless communication, to develop a wearable system that helps users assess their physical and mental health before outpatient visits [235]. They identified patterns correlating collected data with patient-reported questionnaire responses, assisting doctors in diagnosis. Monitoring is particularly crucial for depression patients with cardiac conditions. For example, patients with coronary heart disease (CHD) who do not respond to depression treatment have a higher mortality risk compared to responders. Carney et al. used continuous ECG monitoring to track patients, observing changes in nocturnal HR and HRV before and after treatment [236].

**Figure 11:**
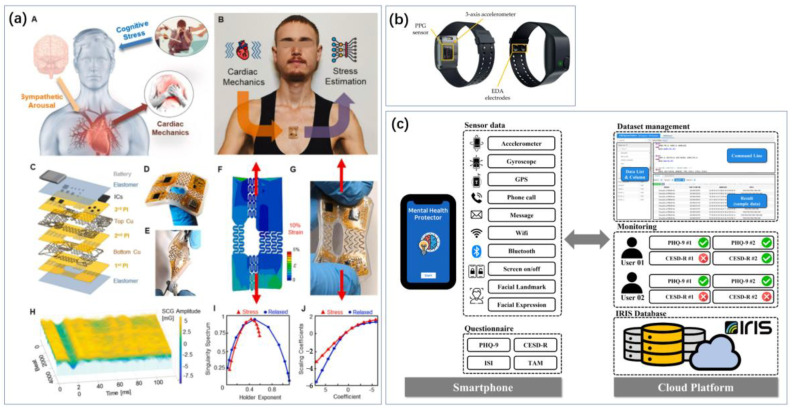
Heartbeat and motion sensing focuses on depression and stress monitoring. (**a**) A vibration sensor attached to the sternum captures changes in cardiac mechanical properties (such as changes in blood pressure, heart rate and other parameters) to estimate the psychological stress of the wearer [240]. Red arrows indicate the direction of stretching. Reproduced under the terms of the Creative Commons Attribution License, Copyright 2023 by the authors, published by Elsevier B.V. (**b**) Monitoring HRV and GSR signals through a wristband sensor to assess the driver‘s psychological stress. Reproduced under the terms of the Creative Commons Attribution License, Copyright 2023 by the authors, published by MDPI AG; (**c**) A depression prediction system based on multi-modal sensor data and machine learning algorithm in smart phones [242]. Reproduced under the terms of the Creative Commons Attribution License, Copyright 2020 by the authors, published by MDPI, Basel, Switzerland.

Technologies for monitoring heart rate using everyday wearable devices are relatively mature [237]. Jo et al. used a photoplethysmography (PPG) sensor in a smartwatch to monitor HRV [238]. Among the HRV indicators investigated, the RMSSD, SDNN, SDSD, LF, and LF/HF ratio were significantly correlated with PHQ-9 scores, with all HRV indicators showing a negative correlation with self-reported clinical symptoms. Cajal et al. attempted to obtain PPG signals using a smartphone camera and identified the overestimation of high-frequency HRV components as a limitation of smartphone PPG in depression monitoring. Vaishali et al. further reduced the impact of heart rate sensors used for depression monitoring by calculating the heart rate from facial video input [239]. Using the Euler video magnification algorithm, heart rate was calculated from facial video, offering the advantage of non-contact measurement. The classification accuracy of “depressed” versus “non-depressed” categories ranged from 96% to 100%. Monitoring of driver pressure by combining signals from PPG and ECG to monitor HRV was realized by Costantini et al. (Figure 11b) [237].

Additionally, heart rate can be measured by sensing the arterial pulse waves generated by the cyclic contraction and relaxation of the heart. Woon-Hong Yeo et al. utilized highly conformal and stretchable patches to capture minute mechanical vibrations on the sternum for monitoring heart rate (Figure 11a) [240]. They applied wavelet transform processing to the heartbeat signals for psychological stress monitoring. Se-kyoung Youm et al. used piezoelectric film sensors to sense the ear pulse wave (EPW) and convert it into electrical current, estimating heart rate based on pressure changes on the ear canal surface [241]. However, pressure variations and the peak height of pressure waves can be affected by body movement and the wearing of ear-mounted devices, introducing errors in heart rate estimation. In contrast, ECG data provide clear information about the depolarization of the left and right ventricles of the heart and have quantitative correlations with clinical standards. Ultra-thin elastic electrodes can capture high-fidelity physiological electrical signals while maintaining high conformability.

Similar to heart rate, breathing is also relevant. Changes in breathing patterns, such as increased or irregular breathing frequency, are associated with anxiety and stress states, and depression patients often exhibit these symptoms with individual variability. Monitoring heart rate and breathing can help detect emotional fluctuations, increased anxiety, and sleep disturbances on time, providing early warning signals and enabling prompt intervention [243]. Breathing exercises can also improve anxiety without medication [243]. Pinho et al. used Doppler radar to acquire respiratory signals from subjects watching videos eliciting different emotions (fear, happiness, and neutrality) [244]. They employed support vector machines, K-nearest neighbors, and random forests to classify the signals, achieving approximately 70% emotion recognition accuracy and demonstrating the feasibility of non-contact respiratory signal measurement for characterizing emotional stress. Given that heart rate and breathing are commonly used in sleep and exercise monitoring, current technologies are highly advanced. Although many sensors are not specifically designed for depression monitoring, they can be incorporated into wearable systems for depression, as discussed in Section 3.5.

### 3.2. Limb Movement Sensing

Monitoring limb movements can reflect the daily activity levels and movement patterns of individuals with depression. Research indicates that due to basal ganglia activity and psychomotor deficiencies, patients with depression exhibit significantly reduced stride length and walking speed compared to healthy individuals, prolonged stance time on both legs, decreased arm swing, reduced vertical head movement, and increased lateral head sway. Monitoring limb movements can provide insights into the patient’s activity levels and patterns, with decreased activity potentially signaling worsening depressive symptoms or even suicide risk. Moreover, the side effects of medication and the impact of depression on the nervous system may lead to reduced body coordination and uncontrolled hand tremors. Long-term tracking of these data can establish personalized health baselines, detect abnormal trends, and aid in the early identification of symptoms for timely intervention.

Currently, common clinical motion capture systems often use cameras to collect motion images, requiring subjects to wear multiple sensors (reflective markers). Additionally, force plates can extract ground reaction forces in the x, y, and z directions during walking to analyze gait [245]. However, these systems are generally non-wearable, imposing limitations on subject movement and making real-time, on-site motion recognition challenging.

Common wearable motion sensors can be broadly categorized based on their principles into pressure sensors, strain sensors, accelerometers, gyroscopes, magnetometers, and electromyography (EMG) sensors. Lau et al. developed a self-powered strain sensor based on graphene oxide-polyacrylamide (GO-PAM) hydrogel for monitoring gait and other human movements, which also functions as a triboelectric nanogenerator (TENG) to harvest mechanical energy. A wearable insole sensor system, integrating a data processing module and PC interface, uses artificial neural networks (ANNs) to achieve recognition rates of 99.5% and 98.2% for everyday and pathological gait, respectively, demonstrating its potential for depression auxiliary diagnosis [246]. Tranberg et al. developed a system with inertial sensors to quantify gait symmetry and normality, evaluated in a laboratory setting using 3D motion measurement results [247]. Rong Zhu et al. proposed a motion capture method using a single device, integrating a micro three-axis flow sensor and micro three-axis inertial sensor, allowing for the accurate measurement of 3D movement speed, acceleration, and posture angles of the limbs during daily activities, vigorous exercise, and prolonged exercise [248]. A flexible sensor-based goniometer is also used to measure angles at different joints, such as the ankle, knee, or hip. Zhonglin Wang et al. developed a self-powered piezoelectric electronic skin (PENG) that generates current signals based on ankle joint movements to recognize various activities such as stepping, jumping, and squatting [249]. Additionally, various motion recognition and feature extraction algorithms [250] have been researched based on different sensor-type combinations, and wearable motion recognition devices have been widely applied in monitoring and rehabilitation for Alzheimer’s disease, hemiplegia, epilepsy, and related mental disorders.

Gait analysis typically involves measuring average basic gait cycle features, such as walking speed, cadence, stride width, step length, and stride length [251]. Bin Hu et al. proposed a multimodal gait analysis method for depression detection that combines skeletal and contour modalities [252]. Using cameras to capture depth videos and limb joint coordinates, along with depression self-assessment scales, they created a skeletal feature set for depression. A multimodal fusion model was proposed, achieving an accuracy of 85.45% in a dataset of 200 students (including 86 students with depression) based on multi-view contour features and directly visualizing gait differences between depressed and non-depressed individuals through skeletal visualization. This method demonstrates that gait is an effective biomarker for depression detection. Tognetti et al. developed a smart wristband that integrates a triaxial accelerometer, temperature sensor, and PPG sensor, capable of acquiring motion information while mitigating some motion artifacts affecting PPG light sources and electrodermal activity (EDA) electrodes [253]. This system can monitor autonomic nervous system stress responses real time and related neurological disorders through multimodal signals.

### 3.3. Bioelectrical Sensing

EDA is a key indicator for assessing stress arousal and cognitive states by sensing the sympathetic nervous system [254]. All endocrine sweat glands in the body respond to emotions, cognition, and temperature, with the palms and soles exhibiting heightened sensitivity to emotional stimuli. Thus, certain characteristics of skin sweat can indirectly reflect psychological stress. Skin conductance (GSR) and skin potential are the primary physical properties measured in EDA. Short-term changes in skin conductance are often related to specific emotional stimuli, making the phase components of GSR useful for quantifying psychological stress [255]. Hidalgo-Mazzei et al. compared EDA signals between bipolar disorder patients and healthy individuals, finding that both tonic and phasic EDA components were reduced in bipolar depressive patients [256]. However, these EDA parameters returned to normal levels following symptom relief, indicating that EDA is not only a physiological marker for bipolar depression but also correlates with treatment levels.

**Figure 12:**
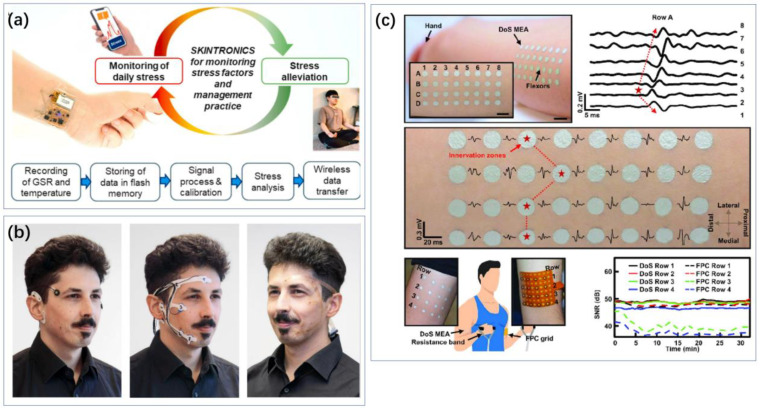
Various types of wearable bioelectrical sensing. (**a**) Skintronics integrates GSR electrodes, skin temperature sensors and small batteries for wireless monitoring and management of human stress levels [259]. Reproduced under the terms of the Creative Commons Attribution License, Copyright 2020 by the authors, published by Elsevier B.V. (**b**) Flex-printed pre-gelled sensor arrays designed for sleep electroencephalography (EEG) acquisition [267]. Reproduced under the terms of the Creative Commons Attribution License, Copyright 2022 by the authors, published by frontiers. (**c**) Large-area, high-density electromyography electrode arrays directly drawn on the skin, suitable for various muscle structures [268]. Reproduced under the terms of the Creative Commons Attribution License, Copyright 2023 by the authors, published by Oxford University Press on behalf of National Academy of Sciences.

Common GSR sensors generally detect changes in electrical activity caused by sweat gland activity through electrodes [257]. The quality of these signals is highly dependent on electrode characteristics and the skin area, such as mechanical stability, viscosity, and electrolyte concentration, matching the salt concentration of skin sweat (as GSR signals are influenced by skin hydration), as well as interference from electrode artifacts [258]. Kim et al. analyzed users’ stress levels using wearable GSR signals (Figure 12a) [259]. The most basic measurement electrodes are Ag/AgCl gel electrodes, which effectively reduce the electrode–skin interface impedance, thus improving signal quality [260]. However, gel materials have limited breathability, and long-term wear may affect skin comfort [261]. Additionally, as the gel layer degrades, the impedance at the electrode–skin interface increases, leading to greater signal distortion from motion artifacts and noise, which significantly degrades EDA signal quality [260]. Recent developments have introduced various alternative electrode solutions, such as highly breathable textile electrodes, low-impedance and high-stability dry carbon/salt adhesive electrodes [262], elastic electrodes based on conductive polymer-based foams [263], and electrodes made from screen-printed silver, carbon, or conductive polymer inks [264]. Based on electrode signals, various algorithms have also emerged for feature extraction, motion artifact identification, and error correction in EDA signals [265,266].

The brain, as the central nervous system, has a close association with depression. Tian et al. designed a flexible wearable three-electrode EEG system that achieves a 90.70% accuracy in depression recognition [269]. Liu et al. developed a system for the real-time diagnosis of depression using EEG, which plays music therapy in response to emotional changes, providing more timely emotional intervention [270]. Kaur et al. utilized EEG for source localization of depression, which was consistent with results from magnetoencephalography [271]. Rahman et al. focused on combining near-infrared and EEG monitoring to enhance depression recognition capabilities [272]. However, there is a lack of reports on wearable monitoring for home use by patients.

Veeranki et al. compared various algorithms used to process nonlinear signals that have extracted more information related to emotions in EDA signals [273]. Improved symbolic aggregate approximation (isaxEDA) achieved the best results with an f1 score of 65% using an SVM classifier. This study supports the applicability of nonlinear EDA signal processing to improve emotion recognition, which can be used to detect mental health conditions such as anxiety and depression. The acquisition of EEG signals involves data from multiple electrodes in the brain, and the weights are not necessarily constant across channels. Shen et al. proposed an adaptive channel fusion method based on an improved focal loss (FL) function, which can be optimized by the proposed adaptive channel fusion framework to optimize the channel weights [274]. Experimental results on two EEG signal datasets show that the proposed channel fusion method can improve the classification performance. While many studies have focused on depression algorithms based on EEG signals, this review does not systematically address these studies due to the lack of sensor-specific innovations. Nevertheless, these studies offer the potential for improving recognition effectiveness and reducing the number of electrodes required [274,275,276].

Lastly, studies on measuring heart rate from myocardial electrical signals also fall under this section. However, due to the physiological phenomenon primarily related to heartbeat, it is covered in Section 3.1, while the study on limb movement is located in Section 3.2. Many bioelectrical sensing studies that are not specific to depression also have the potential to be introduced as depression. For example, the wire-power system designed by Choi et al. releases electrical stimulation for cardiac pacing, and electroshock therapy is one of the ways to alleviate depression [277]. There have been a number of studies that have focused on work such as the comfort of wearable electrodes (Figure 12). These electrodes are often not developed for depression but only have the generality of electrical signal acquisition and, therefore, are not reviewed in this paper.

### 3.4. Sleep and Circadian Monitoring

Depression patients frequently experience sleep disturbances. Research has confirmed that depression can lead to circadian rhythm disorders [278,279]. Additionally, the degree of misalignment in circadian rhythms is correlated with the severity of depressive symptoms [280]; as depressive symptoms improve, circadian rhythms tend to return to normal [281]. Insomnia patients without depression are twice as likely to develop depression compared to individuals without sleep difficulties [12].

Robillard et al. used activity monitors to measure sleep states in 342 subjects [282]. They found that, compared to healthy individuals, patients with anxiety, bipolar disorder, and other forms of depression had significantly later sleep onset times, longer sleep durations, lower sleep efficiency, and delays in circadian activity rhythms. Migliorini et al. compared sleep records between healthy adults and bipolar disorder patients using a T-shirt integrated with ECG and respiration sensors along with accelerometers [283]. Their results showed differences in heart rate reduction in bipolar disorder patients and an increase in the percentage of non-rapid eye movement (NREM) sleep.

The gold standard for objectively assessing sleep characteristics is polysomnography (PSG), which integrates data from EEG, electromyography (EMG), electrooculography (EOG), electrocardiography (ECG), respiration sensors, and blood oxygen saturation sensors. Clinical diagnoses are made based on these integrated results. However, this method requires 8–12 h of testing in a controlled laboratory setting, which is relatively inconvenient for assessments. Recently, various wearable devices for sleep monitoring have emerged, often incorporating combinations of accelerometers [283], heart rate sensors [284], audio-based respiration sensors [285], and neuroelectrical signal electrodes [286]. Different sensor combinations can lead to varying identification accuracies. There is also a gap between the quality of data acquisition in consumer electronics and clinical sleep polysomnography. Hu et al. investigated the problem of missing data in wearable devices during data acquisition and proposed a systematic classification model for depression, combining five classification models, namely SVM, KNN, LR, CBR, and DT, with an improvement of 28.56% in terms of MAE as a metric gain [287]. Nonetheless, there is still a lack of dedicated wearable sleep monitoring sensors, and, often, multiple physiological signals need to be simultaneously monitored to achieve accurate sleep assessments.

Moreover, compared to sleep parameters, 24 h circadian rhythm parameters show a stronger correlation with depression, such as skin temperature, core temperature, melatonin levels, and activity levels [280,288]. Depression patients exhibit bluntness and phase shifts in core body temperature curves [289]. Since core body temperature typically requires invasive measurement methods, skin temperature has become an alternative measurement method in recent years. Emotional fluctuations and stress affect blood circulation and sweat gland activity; during periods of low mood, blood flow to the skin decreases, potentially lowering skin temperature, while anxiety activates the sympathetic nervous system, increasing blood flow to the skin and raising skin temperature [290]. Hegerl et al. used activity monitors to measure skin temperature, thermal flux, and current response, confirming that the relative amplitude of skin temperature curves is significantly reduced in depression patients, with some improvement observed with antidepressant treatment [291]. T. Singer et al. introduced thermal infrared imaging analysis in an experimental setting with 15 subjects studying social stress [292]. They found significant temporal variations in nasal tip temperature, perioral temperature, and fingertip temperature, with fingertip temperature showing a strong correlation with stress. Given that skin temperature can be influenced by environmental temperature and skin conditions, many studies have integrated skin temperature sensors with other physiological signal sensors for auxiliary depression diagnosis [293,294,295]. For example, Wang et al. developed a ring-shaped wearable device integrating EDA sensors, heart rate sensors, skin temperature sensors, and accelerometers to assess psychological stress levels and emotional recognition [296]. Combined with a backend IoT platform, this system achieved a maximum accuracy of 83.5%.

### 3.5. Daily Behavioral Monitoring

In addition to the more obvious indicators above, a person’s daily activities, language, socialization, and other behaviors may all contain depression-related information. While some medical devices may cause some stress to the user, much of the daily behavioral information can be captured in a way that is not obviously perceived by the patient, helping to monitor the patient’s depression in its most natural and real state.

Price et al. analyzed levels of depression based on activity information collected by wristbands [297]. Language is also rich in information. Niu et al. took the collected voice/video information of the subjects and carried out analysis using a novel spatio-temporal attention (STA) network and a multimodal attention feature fusion (MAFF) to achieve better ratings [298]. Soto et al. showed good application by analyzing facial expression data and comparing depression analysis through visual information and language models [299].

**Figure 13:**
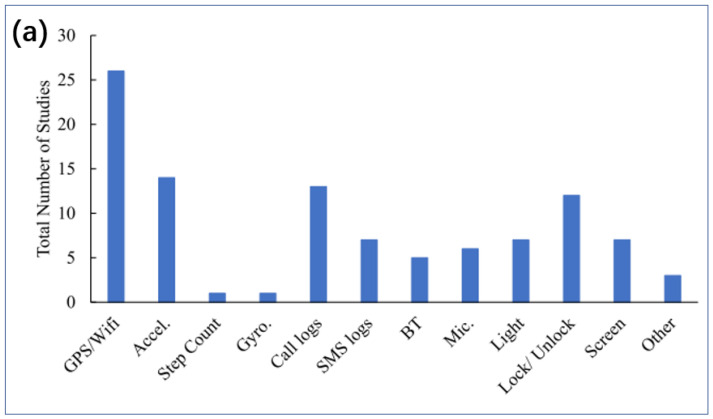

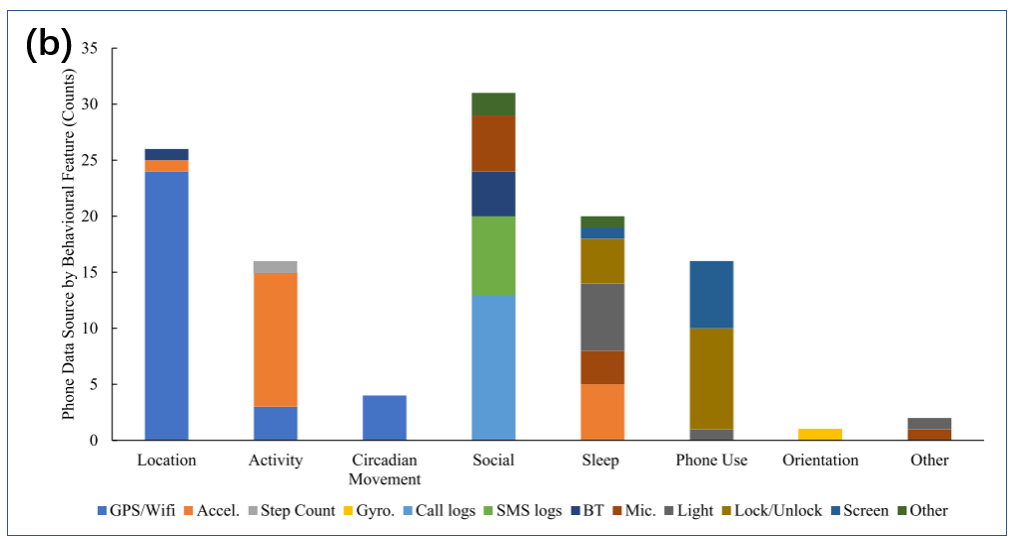
(**a**) Total number of studies using each source of phone data. Note. Accel. = Accelerometer; Gyro. = Gyroscope; BT = Bluetooth; Mic. = Microphone. (**b**) Number of studies using each sensor type to infer high-level behavioral features. Accel. = Accelerometer; Gyro. = Gyroscope; BT = Bluetooth; Mic. = Microphone [301]. Reproduced under the terms of the Creative Commons Attribution License, Copyright 2024 by the authors, published by Elsevier Ltd.

Existing smartphones integrate motion-related sensors, such as accelerometers, GPS, Hall sensors, etc., which can analyze the various movements of a person while holding or carrying them, and screen-lighting times, etc., can also reflect activity (Figure 13). Hong et al. developed big data algorithms based on this and achieved the successful prediction of 93.75% of patients with depression in the sensing data of the daily use of cell phones [242]. Ho et al. added monitoring arranged within a living scenario based on electronics for personal use, proposing REMONI, an autonomous remote health monitoring system integrating a multimodal large language model (mllm), the Internet of Things (IoT), and wearable devices [300]. Components are developed from models capable of detecting and recognizing patient activity and emotions while responding to queries from healthcare professionals.

The development of big models gives more possibilities for such depression diagnosis based on behavioral, voice, and video big data. This includes the addition of GPS, WiFi access, and other signals to motion data for richer analysis, collectively known as digital phenotyping [301]. Since most sensors are already integrated into consumer electronics, related research has focused more on the design of algorithms, and, often, it is machine learning algorithms rather than the traditional idea of reducing marker concentrations and physiological signal strengths. More studies are summarized in Section 3.6.

The development of this type of technology has undoubtedly facilitated the daily monitoring and diagnosis of patients with depression and can be analyzed from information on daily activities without the cooperation of the patient. However, this may also bring some ethical challenges, such as whether schools can determine students’ depression and force them to be treated through daily monitoring, students’ homework, and other information, whether enterprises can identify employees with depression through chat records in the company, and whether this will lead to difficulties in their job search and promotion. Currently, many cases of depression are not detected in time, or the patients themselves have no intention of obtaining treatment; does this mean that the government, guardians, etc., have the right to carry out the help they think they need? At a time when more and more sensors and big models are integrated into our surroundings and consumer electronics are also evolving, this may become a new ethical issue.

### 3.6. Integrated Wearable System

Previous research has demonstrated a strong correlation between physiological information—such as heart rate variability, blood pressure, eye movements, skin conductance, muscle activity, skin temperature, and brain electrical signals—and psychological stress [302]. In recent years, advancements in wearable biosensors and wireless communication have facilitated the gradual adoption of medical devices for monitoring heart rate, arterial blood pressure, arterial oxygen saturation, respiration rate, and body temperature. Wireless, real-time, and customized monitoring and auxiliary diagnosis for depression have become feasible. Long-term monitoring of relevant physiological and behavioral parameters allows for a more comprehensive assessment of depression patients’ treatment outcomes, providing early warning mechanisms to prevent the worsening of depressive symptoms or extreme events.

Many existing wearable devices integrate GSR electrodes with other sensing systems for multimodal physiological signal monitoring [256], facilitating the assessment of mental health conditions such as depression. Myoungho Lee et al. integrated GSR electrodes with pulse-wave sensors and corresponding signal processing systems into smart fabric gloves for evaluating emotional stress through sleep monitoring [303]. N. Sebe et al. used commercial GSR sensors, EEG sensors, ECG sensors, and facial activity data collected while users watched emotional movies to build a multimodal emotional recognition database [304]. Woon-Hong Yeo et al. reported a wireless, portable, real-time skin conductance and temperature sensor based on soft nanomembrane electrodes, rechargeable batteries, and flexible circuits [240]. This system calibrates temperature-induced errors to accurately measure stress and uses skin conductance signals to characterize mental stress levels in various contexts such as daily office work, household chores, and meditation. Numerous studies have confirmed that wearable sensors can monitor the presence of depressive symptoms. Yu et al. extracted average amplitude, slope, and standard deviation information from GSR signals of depression patients and developed identification algorithms that achieved over 70% accuracy and sensitivity in identifying severe depression [231]. Mischoulon et al. used a smart wristband and smartphone embedded with GSR sensors, temperature sensors, heart rate (HR) sensors, and a three-axis accelerometer to monitor the movement, sleep, and social characteristics of 41 depression patients [305]. They found that combining physiological and movement signals using machine learning algorithms provided more accurate assessments of depression severity.

Picard et al. continuously recorded ECG, EMG, skin conductance, and respiration signals from drivers in real driving scenarios to distinguish between three levels of driver stress, achieving a high success rate of 97% [306]. Their results indicate that heart rate and skin conductance signals are most closely related to driver stress levels in driving contexts but may require additional physiological signals in other contexts. Hermens et al. proposed a method for predicting mental stress using nine physiological features from four types of signals: ECG from the upper trapezius, respiration, skin conductance, and surface EMG [307]. This method achieved a classification rate of 74.5% in office work scenarios with three different stress sources. While this approach uses multimodal physiological signals to predict mental stress across various scenarios, it may lead to cumbersome wearable systems, and its identification rate needs improvement. Gutierrez-Osuna et al. combined respiratory power spectral density and heart rate variability information to estimate psychological stress [308]. Their sensor system included a heart rate monitoring wristband, a chest strap with pressure sensors to monitor respiration, AgCl electrodes for skin conductance monitoring on the index and middle fingers, and EMG electrodes for monitoring trapezius activation. Their results showed that specific feature extraction algorithms could successfully distinguish between depressive and non-depressive mental states with a success rate of 81%. This solution aims for a simplified and non-intrusive wearable system design while achieving high classification accuracy with minimal sensors.

Existing wearable biosensors can integrate multiple flexible devices into textile garments or body surfaces to enable multimodal information monitoring. Additionally, microelectromechanical system (MEMS)-based miniature motion sensors (such as accelerometers, gyroscopes, and magnetometers) are widely used to measure human activity-related signals. The feasibility of using wearable devices for depression assessment through sensor integration, communication systems, and signal processing systems has been validated. Researchers tracked activities using smartphones for 1002 subjects and monitored multimodal physiological signals such as step count, sleep, and heart rate using wearable devices. Based on this large dataset combined with clinical stress assessment questionnaires, typical biomarkers for depression, such as sleep quality, heart rate, stress, daily activity, and exercise, were identified [309]. This indicates that adverse mental states such as high depression, anxiety, and psychological stress are significantly correlated with specific physiological responses. Moreover, studies have increasingly used wearable devices to monitor physiological information across various scales, populations (e.g., university students [310], drivers [311], pregnant women [312]), contexts (e.g., leisure [313], work [307]), and locations (e.g., office [307], laboratory [314], outdoors [302]) to obtain health status and psychological stress indicators. For small-sample datasets, shallow machine learning models and feature extraction methods can typically provide classification information. For larger, more diverse, and varied datasets, more complex deep learning models are often required to derive psychological stress levels from wearable physiological information. Dai et al., based on a public dataset including 8996 participants and 1247 diagnosed with mental disorders, developed an end-to-end deep learning model, Wearnet, using a smart wristband with embedded inertial and heart rate sensors to monitor total steps, calorie consumption, average heart rate, and activity time [315]. They employed transformer encoders and convolutional neural networks to study depression and anxiety detection using commercial wearable activity trackers. The system demonstrated high recognition capability (AUROC of 0.717), further confirming the feasibility of monitoring mental disorders using commercial wearable devices in large populations.

Finally, long-term monitoring systems may also include biochemical sensing. Although there are fewer applications of the previously mentioned markers that are strongly associated with specificity, monitoring systems that include biochemical sensing have been made. Xu et al. reported an electronic skin for stress response assessment that non-invasively monitors three vital signs (pulse waveform, galvanic skin response and skin temperature) and six molecular biomarkers in human sweat (glucose, lactate, uric acid, sodium ions, potassium ions and ammonium) [230,316]. With the help of a machine learning pipeline, the platform can differentiate three stressors with an accuracy of 98.0% and quantify psychological stress responses with a confidence level of 98.7%.

**Figure 14:**
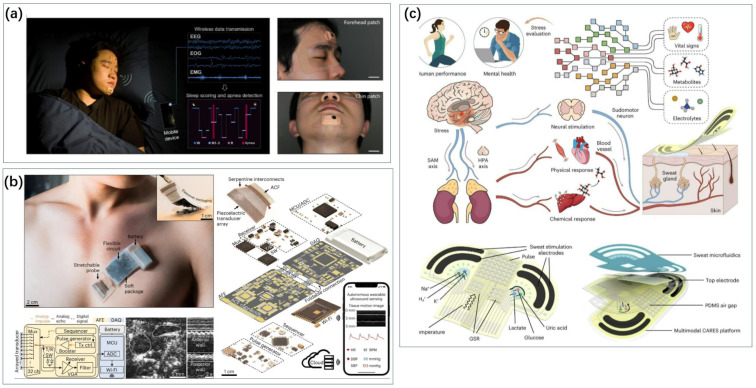
Types of multimodal systems. (**a**) The skin patch with integrated EOG, EEG and EMG electrodes was used to evaluate sleep quality and apnea, and showed similar performance as PSG in control experiment [286]. Reproduced under the terms of the Creative Commons Attribution License, Copyright 2023 by the authors, published by American Association for the Advancement of Science. (**b**) A fully integrated autonomous wearable ultrasound patch system capable of continuously monitoring cardiac signals such as central blood pressure, heart rate, and cardiac output for 12 h, without being affected by the wearer’s movements [230,316]. Reproduced with permission, Copyright 2023 Springer Nature America, Inc. (**c**)The wearable flexible patch can simultaneously monitor pulse waveform, GSR, skin temperature, sweat metabolites (glucose, lactic acid and UA) and electrolytes (Na+, K+ and NH4+) in real time, to quantify the level of psychological stress [230]. Reproduced with permission, Copyright 2024 Springer Nature Limited.

There is a wealth of research related to depression because of the abundance of test objects and sensing technologies related to depression and the fact that there is currently no uniform gold standard scheme for sensing in the industry. Overall, the integration of sensing systems can be divided into three categories. One is the integration of different signals with the same sensing principle, such as the collection of EEG, EMG, ECG, etc., with electrodes, which is used to comprehensively determine depression (Figure 14a) [286]. The second is to use multiple methods to measure the same physiological activity, such as accelerometers and electrodes, together to measure the heartbeat and, thus, more accurately monitor a particular depression-related indicator (Figure 14b). The third is integrated sensing, i.e., the comprehensive integration of motion, bioelectricity, biochemical sensing, etc., to monitor depression from multiple perspectives (Figure 14c) [230,316].

This review summarizes current advances in wearable depression monitoring systems (Table 4). The integration of more sensors may face difficulties, especially since most consumer electronics currently come in the form of watches, rings, etc., whose space is more limited. More sensors such as gait, respiration, EEG, etc., may need to be integrated into products such as hair bands, insoles, and clothing. In addition, due to the different habits of people using electronic products, the weights between different indicators may need to be adjusted, and even missing data often occur. Some of the previous algorithmic research may be helpful in this regard to better utilize data that do not come from the same product.

**Table 4:** Integrated wearable depression monitoring system.

Sensors	Output	Indicators	Feature	Ref.
EOG sensor, ECG sensor. GSR sensor, breathing sensor	stress levels	97% classification rate	heart rate and skin conductance signals are most closely related to driver stress	[306]
ECG sensor, breathing sensor, skin conductance, and surface EMG sensor	stress levels	74.5% classification rate	Use a variety of pressure sources	[307]
total steps, calorie consumption, average heart rate, and activity time	stress levels and emotional recognition	AUROC over 0.717	The system confirmed the feasibility of monitoring mental disorders using commercial wearable devices in large populations.	[315]
EDA, heart rate, temperature, and accelerometers	stress levels and emotional recognition	83.5% classification rate	combined with a backend IoT platform	[296]
temperature, blood pressure, heart rate, GSR	Correlation with salivary cortisol levels	\	LF/HF ratio of HRV and skin temperature may be good indices for the assessment of life stress	[317]
ECG sensor, EMG sensor, GSR sensor	driving stress levels	85.3% classification rate	build a model that can identify drivers’ stress accurately in real time	[311]
HRV sensor, GSR sensor, temperature sensor	Changes in physiological signals before and after experiment	\	GSR can be used as a pressure marker	[318]
GSR, heart rate	stress levels	99.5% classification rate	Psychological stress can be monitored using only two physiological signals	[319]
GSR, HRV, respiration rate	stress levels in talk	\	partners were more stressed when speaking with friends than to one another about relationship challenges	[320]
EEG, GSR, respiration rate	stress levels in VR	85% classification rate	VR video games can alleviate stress.	[321]
ECG, EMG, HRV, GSR, temperature	depression levels	93.5% classification rate	Multi-mode signal helps improve the recognition accuracy	[322]
EEG, HRV, GSR, eye tracking data	mental fatigue and stress	\	Assessment of mental fatigue and stress on electronic sports players with data fusion	[323]
heart rate and its variability (HRV), pulse arrival time, GSR, blood oxygenation level (SpO_2_), respiratory rate	sympathetic nervous system activities	\	A novel wearable biomedical device enabling the synchronous acquisition of PPG, ECG, GSR, and motion signals directly on the fingers	[324]
GSR, HRV	stress levels in workspaces	92.5% classification rate	Local maxima and minima (LMM) from HRV and GSR sensors can improve the detection performance	[325]

## 4. Discussion

Depression is a growing threat to people’s health and public health. Its causes, manifestations, and treatment outcomes are highly individualized, and patients often have long treatment cycles. There is a great need for better personalized treatment through convenient and inexpensive sensing technologies. Early diagnosis of the development and recurrence of depression, early warning of extreme mood or somatization symptoms, and assistance in analyzing etiology are necessary. However, the lack of a gold standard makes it difficult to have a unified technology path and measurements for sensing technologies regarding depression. Many biochemical sensing technologies may only be effective for patients with depression of a specific etiology or manifestation, and sensing technologies for some of the markers themselves are still scarce. Comprehensive biochemical monitoring can involve dozens of different markers, which is a great challenge for both the sensing technology itself and the algorithms. The way forward may still be to look for solutions that can provide personalized treatment for patients with a single or small number of markers, underneath the established diagnosis. The sensing technology of bioelectricity and motion has benefited from its migratory nature for the monitoring of many diseases, sleep, motion, etc., and the sensors themselves are more richly researched. Existing commercialized solutions can help with testing for stress or make some distinction between depressed individuals in generalized data. However, a depression monitoring system that relies entirely on wearable objective sensing results, without relying on subjective evaluations and hospital tests, needs further research. This will require a richer set of portable or wearable sensing technologies, along with the co-development of corresponding datasets and algorithms. It is difficult to carry out research on biosensors, especially systems, when there is still so much unknown at the biological level about the complete physiological processes, markers, and disease relationships in depression. The development of machine learning, especially large models, has given a boost to depression diagnosis based on information about activities such as daily speech but has introduced potential ethical issues. The same line of thinking could be used to help further applications of biochemical sensing; where the association between individual chemical markers and depression is limited, perhaps larger databases could be built.
